# Adaptive Arc-Voltage Control for Narrow-Gap Pulsed TIG Welding Based on Feedback Reconstruction and Effective-Error Identification

**DOI:** 10.3390/s26206484

**Published:** 2026-10-13

**Authors:** Haiping Fan, Lei Guo, Yunda Duan, Weihua Liu, Yuefeng Chen, Minjun Peng

**Affiliations:** 1 College of Nuclear Science and Technology, Harbin Engineering University, Harbin 150001, China; fhp_1@hrbeu.edu.cn (H.F.); heupmj@hrbeu.edu.cn (M.P.); 2 China Nuclear Industry Fifth Construction Co., Ltd., Shanghai 201506, China; gl1999@hrbeu.edu.cn (L.G.); duanyunda@cnec5.com (Y.D.); cyfwad@163.com (Y.C.); 3 College of Mechanical and Electrical Engineering, Harbin Engineering University, Harbin 150001, China; 4 School of Materials Science and Engineering, Shanghai Jiao Tong University, Shanghai 200240, China

**Keywords:** narrow-gap welding, pulsed TIG welding, adaptive arc-voltage control, feedback reconstruction, effective-error identification, fuzzy PID, multi-objective optimization

## Abstract

Reliable arc-length regulation is essential in narrow-gap pulsed tungsten inert gas (TIG) welding because restricted groove clearance makes small torch-height errors capable of destabilizing the arc and reducing process repeatability. However, pulse switching, wire–pool interaction, and acquisition noise generate voltage transients that conventional arc-voltage control (AVC) may misinterpret as geometric deviations, whereas strong filtering delays genuine drift correction. This study proposes a reconstruction–decision–adaptation architecture. An experimentally calibrated actuator–arc model supports adaptive Kalman filter (AKF)-based feedback reconstruction; an amplitude–duration–area criterion fixed from open-loop statistics rejects brief disturbances; and only identified effective errors drive online fuzzy-PID gain adaptation. Using fixed decision thresholds, NSGA-II jointly tunes estimator and controller parameters based on voltage RMSE and cumulative actuator travel. Across 20 simulation seeds, the tuned configuration reduced voltage RMSE and ramp-disturbance error by 15.8% and 51.0%, respectively, while travel decreased by 1.9%. Physical welding tests demonstrated improved voltage regulation while maintaining actuator activity: the 13.0 V comparison reduced RMSE by 19.7% and adjustment events by 8.3%, while six additional records at three prescribed voltages showed consistent mean voltage regulation within the tested conditions.

## 1. Introduction

Narrow-gap tungsten inert gas (TIG) welding is attractive for thick-walled pressure vessels, pipelines, and other high-integrity structures because the reduced groove volume lowers filler-metal consumption, heat input, and welding distortion [1]. These production benefits are accompanied by a demanding control problem. The restricted groove provides little tolerance for vertical torch-position error, while thermal deformation, dimensional variation, electrode consumption, and layer-to-layer geometry change the torch–workpiece distance during a bead. A slowly increasing distance can reduce heat concentration and shielding effectiveness; an excessively short distance can destabilize the arc or cause electrode contamination. Maintaining arc length is therefore not merely a motion-control task but a sensing problem that directly affects process repeatability.

Automated welding systems can estimate geometric and process states from vision, laser profiles, acoustic emission, electrical variables, and combinations of these modalities [2]. Vision provides rich spatial information and has enabled simultaneous torch-center and height detection in narrow-gap welding [3], but optical systems require additional calibration, installation space, and protection from arc radiation, fumes, and contamination. Arc sensing instead treats the welding process itself as the sensor. In constant-current GTAW, a change in arc length produces a corresponding change in voltage, enabling automatic voltage control (AVC) without an external sensor head. This principle has already supported adaptive motion in sensor-enabled robotic welding systems [4] and remains particularly attractive inside a narrow groove where optical access is restricted.

The usefulness of arc voltage, however, does not imply that every measured voltage change represents a correctable height error. Pulsed TIG alternates between current levels, so switching transients and plateau changes are superimposed on the arc-length information. Wire addition and molten-pool dynamics introduce further fluctuations; arc-voltage signals have consequently also been used to recognize droplet-transfer events rather than geometric displacement alone [5]. Moreover, the voltage–length relationship depends on process conditions and can become nonlinear outside a calibrated operating region [6]. The sensor output is thus a mixed signal: it contains the control-relevant evolution of arc length together with pulse-synchronous components, stochastic acquisition noise, and process events that the torch-height actuator should not necessarily follow.

This ambiguity exposes a limitation in conventional AVC design. Direct PID feedback can transform a short voltage spike into a mechanical correction, creating unnecessary travel and reversal. Strong low-pass filtering suppresses such activity but introduces delay and can attenuate the gradual voltage drift caused by real workpiece deformation. Waveform-synchronized sampling improves measurement repeatability by selecting a stable portion of the pulse [7], while Kalman filtering improves arc-state estimation by combining measurements with a dynamic model [8]. Nevertheless, a smoother estimate still does not answer the decision-level question: when is the reconstructed deviation sufficiently persistent and consequential to justify moving the torch? In contrast, different operating conditions may exhibit distinct trade-offs between voltage consistency and actuator activity.

The control objective also contains an inherent trade-off. Aggressive gains and frequent updates can reduce instantaneous voltage error but increase cumulative displacement, backlash crossings, and actuator wear. Conservative tuning reduces mechanical activity at the cost of residual error and weak compensation for slow drift. A weighted single-objective criterion obscures this conflict because its result depends on an a priori weight. Multi-objective evolutionary optimization, particularly NSGA-II [9], can instead expose the Pareto relationship between voltage accuracy and actuator burden. For stochastic welding feedback, however, parameter selection must also remain stable across different noise realizations rather than being optimized for one favorable sequence.

These observations motivate the following research question: can a pulsed-TIG AVC system distinguish sustained, control-relevant arc-length deviation from transient-contaminated voltage feedback, adapt its response online, and improve voltage regulation without increasing mechanical burden? To answer it, this study develops a reconstruction–decision–adaptation framework. Voltage–displacement calibration and open-loop statistics first establish sensor sensitivity and the empirical scale of pulse-related transitions, and an equivalent model links actuator inertia, transmission backlash, torch displacement, and arc dynamics. An innovation-dependent adaptive Kalman filter (AKF) reconstructs voltage and voltage rate; an amplitude–duration–area criterion decides whether the reconstructed deviation is transient or effective; and only the effective error enters a fuzzy PID controller that updates its gains online. The prescribed process target remains fixed, whereas feedback confidence and response gains adapt to the reconstructed process state. Finally, the base PID gains, filter covariances, and fuzzy-adaptation parameters are jointly optimized against voltage RMSE and cumulative actuator displacement, followed by multi-seed simulation, reconstruction analysis, a controller comparison, and multi-condition physical welding validation.

The core contributions are summarized as follows:

1. Model-informed feedback reconstruction. An experimentally calibrated actuator–arc model supports innovation-dependent AKF-based feedback reconstruction.
2. Explicit error-validity decision and online adaptation. A pulse-aware amplitude–duration–area criterion rejects isolated voltage jumps while retaining persistent drift; only this effective error enters the fuzzy PID that updates $K_{p}\left(k\right)$, $K_{i}\left(k\right)$, and $K_{d}\left(k\right)$ online around a fixed process target.
3. Joint estimator–controller tuning. With the experimentally derived error-decision thresholds held fixed, NSGA-II tunes the base PID gains, KF covariances, and fuzzy-adaptation parameters using voltage RMSE and cumulative actuator displacement as competing objectives; representative Pareto solutions are screened over 20 random seeds.
4. Mechanism-specific and system-level validation. Reconstruction delay and jump suppression test the sensing layer; stochastic simulation tests transient rejection and persistent-drift tracking; and controller-comparison plus multi-condition welding records test voltage accuracy, adjustment activity, and response variation under different prescribed operating conditions.

The remainder of this paper is organized as follows. Section 2 reviews sensing, reconstruction, decision, and adaptive-control approaches relevant to pulsed-TIG AVC. Section 3 presents the experimental calibration, plant model, adaptive state estimator, effective-error logic, online gain adaptation, and multi-objective tuning method. Section 4 reports simulation and welding experiments. Section 5 discusses the mechanism, relationship to prior work, and limitations, and Section 6 concludes the study.

## 2. Related Works

### 2.1. Welding Sensors and Arc-Voltage Sensing

Welding automation employs a broad sensing hierarchy. External sensors—including monocular or stereo cameras, structured light, laser profilers, infrared imagers, and acoustic transducers—observe groove geometry, molten-pool behavior, temperature, or process emissions. Recent reviews emphasize that vision sensing provides high-dimensional spatial information but that depth perception, environmental robustness, sensor integration, and real-time processing remain practical bottlenecks [2]. In narrow-gap welding, passive arc imaging can infer both horizontal torch position and vertical height with submillimeter accuracy [3]. Such systems are valuable when lateral seam location and torch pose must be recovered simultaneously, but their optical path and calibration requirements are not always desirable for a compact height-control loop.

Internal or through-process sensing uses electrical behavior already present in the arc. Because voltage varies with arc length under a specified current and process state, AVC can estimate vertical deviation without spatial offset between a separate sensor and the torch. A multi-robot welding and inspection platform demonstrated how measured GTAW voltage can drive real-time vertical path correction [4]. Voltage-based sensing is inexpensive, colocated with the heat source, and immune to direct optical occlusion; however, it is not a direct geometric measurement. The voltage–length relationship must be calibrated for the operating regime. Wang et al. showed that K-TIG voltage can exhibit a nonlinear transition as arc length and arc pressure change [6], illustrating why a sensitivity obtained in one process window should not be generalized without verification.

Electrical signals are also sensitive to phenomena other than torch height. Jia et al. extracted droplet-transfer frequency from wire-filled GTAW voltage, confirming that transfer events leave identifiable structure in the same channel used for AVC [5]. Current switching, electromagnetic interference, pool oscillation, electrode condition, and power-source dynamics add further components. Consequently, the central sensor-design problem is not only to acquire voltage at adequate rate and isolation, but also to distinguish geometric information from other physically meaningful process variations.

### 2.2. Arc-Voltage Preprocessing and State Reconstruction

One class of methods improves the observation before control. Pulse-synchronized acquisition samples or averages voltage within a stable peak-current interval and excludes transition periods. Lei et al. used this strategy for robotic circular-seam arc-length control, obtaining a stable height-related feedback quantity [7]. Its strength is direct use of known waveform timing; its limitation is that disturbances occurring inside the selected plateau remain in the measurement, and useful dynamic information outside the window may be discarded.

Filtering methods operate on the acquired sequence. Moving-average and low-pass filters are easy to implement but impose a fixed frequency-domain compromise between noise attenuation and delay. Model-based estimation instead predicts a latent state and corrects it with the measurement. Wang et al. established a voltage–length relationship for pulsed GTAW and compared Kalman and adaptive Kalman preprocessing for arc-length stabilization, reporting improved stability with adaptive estimation [8]. Kalman filtering is therefore a suitable basis for reconstructing the slowly evolving voltage state, provided that the state model, process covariance, and measurement covariance reflect the physical plant. The present work extends this idea by including actuator backlash and arc dynamics in the estimator interface and by applying derivative action to reconstructed rather than raw voltage.

Preprocessing alone does not define control relevance. A filter can attenuate an isolated peak yet still pass a residual threshold crossing, while excessive smoothing can delay a genuine drift. This distinction motivates a second layer that evaluates the temporal evidence carried by the reconstructed error rather than assuming that every filtered sample should update the actuator.

### 2.3. Arc-Length Control and Error-Driven Updating

Arc-length controllers range from classical PI/PID laws to fuzzy, adaptive, and learning-based schemes. Pulsed-TIG tube-sheet tracking has combined voltage modeling, signal filtering, and fuzzy control to improve online arc-length regulation [10]. Adaptive approaches have also been applied to uneven substrates and wire-arc additive processes, where torch height and deposited-layer height interact [11]. In wire-filled GTAW, transfer-mode recognition and adaptive regulation address the coupling between droplet behavior and voltage feedback [12]. These studies show that nonlinear or time-varying welding conditions benefit from control laws more flexible than fixed-gain PID.

Fuzzy gain scheduling provides an interpretable way to adapt proportional, integral, and derivative action according to error and error rate [13,14]. Yet gain adaptation does not by itself decide whether a deviation originates from geometry or a short process event. If the fuzzy PID is updated at every sample, the actuator can still respond to nuisance fluctuations. A dead band rejects small noise but cannot distinguish a large short spike from a sustained error, and a persistence timer alone can overlook the accumulated effect of a moderate deviation.

Event-triggered control supplies the missing decision perspective: control is updated when a state-dependent condition is met rather than solely according to a periodic clock [15,16]. Data-driven and jointly optimized triggers demonstrate that the event mechanism and feedback performance are coupled [17,18]. Measurement noise remains a central concern because threshold crossings can cause excessive events; robust event-triggered formulations explicitly address this issue [19]. For pulsed-TIG AVC, the practical implication is that the trigger should act on a reconstructed state and should incorporate both instantaneous magnitude and accumulated temporal evidence.

### 2.4. Multi-Objective Controller Tuning and Research Gap

PID gains, fuzzy scaling, filter covariance, and error-validity thresholds affect one another. Increasing filter confidence in the model changes error magnitude and rate; changing PID gains alters displacement and backlash crossings; and smoothing fuzzy corrections changes both transient response and actuator activity. Tuning these blocks sequentially can therefore produce a locally acceptable but globally inconsistent design. NSGA-II offers a well-established mechanism for maintaining a diverse set of non-dominated solutions in competing objectives [9], while Latin-hypercube sampling improves initial coverage of a multidimensional search space [20]. Because measurement disturbances are stochastic, candidate solutions should be evaluated over multiple realizations and revalidated independently [21].

Existing studies have improved arc-voltage sensing, feedback reconstruction, and adaptive control for arc-length regulation. However, the measured voltage in pulsed TIG welding contains both arc-length-related information and non-geometric variations caused by current switching and process disturbances. Therefore, a reconstructed voltage deviation cannot always be directly regarded as a height error requiring actuator correction. Distinguishing control-relevant deviations from transient fluctuations before actuator correction is therefore essential for reliable AVC. Welding studies generally filter voltage and then control continuously; generic event-triggered studies do not encode pulse-specific voltage statistics; and controller optimization often excludes estimator parameters or mechanical adjustment burden. The method developed here closes this interface gap by (i) calibrating voltage sensitivity and transient statistics on the target process, (ii) adapting the measurement confidence of the AKF while reconstructing voltage through an actuator–arc model, (iii) identifying effective errors using magnitude, duration, and area, and (iv) adapting PID gains online from the identified error while jointly tuning the estimator and controller against accuracy and cumulative displacement. In this paper, *adaptive AVC* denotes online estimator-confidence and controller-gain adjustment around a prescribed process-voltage target; it does not denote autonomous scheduling of the target voltage across nominal welding procedures.

## 3. Method

**Figure 1:**
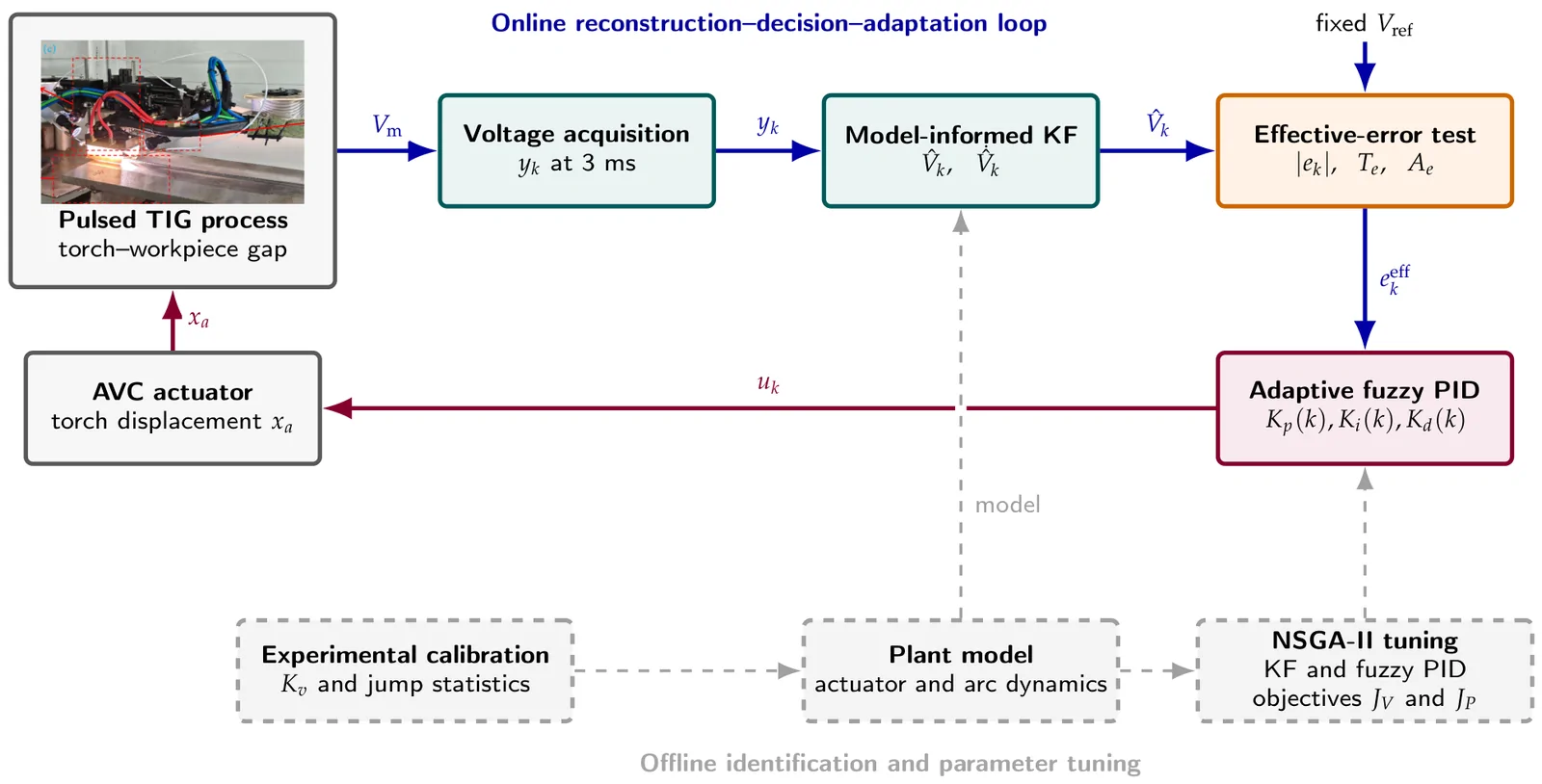
Simplified overview of the proposed adaptive AVC framework. The prescribed process-voltage target is fixed, whereas KF measurement confidence and fuzzy-PID gains are adjusted online. Solid arrows form the online closed loop, and dashed arrows indicate offline identification and base-parameter tuning.

The proposed method consists of three stages: feedback reconstruction, effective-error identification, and adaptive control (Figure 1). First, the calibrated actuator–arc model and AKF reconstruct the control-relevant voltage state. Second, the amplitude–duration–area rule converts the reconstructed deviation into an effective-error decision. Third, the fuzzy PID adapts its gains online and commands torch-height correction only when that decision is active. The physical response closes the loop through the pulsed-TIG process. The lower path supplies the experimentally identified model, fixes the decision thresholds from open-loop statistics, and jointly tunes the estimator and adaptive controller against voltage RMSE and actuator travel. Thus, each block has a corresponding validation target in Section 4: model and threshold calibration, reconstruction fidelity, disturbance rejection and drift tracking, or real-machine voltage–motion performance.

### 3.1. Equivalent Arc-Voltage Model

#### 3.1.1. AVC Actuator and Arc Dynamics

**Figure 2:**
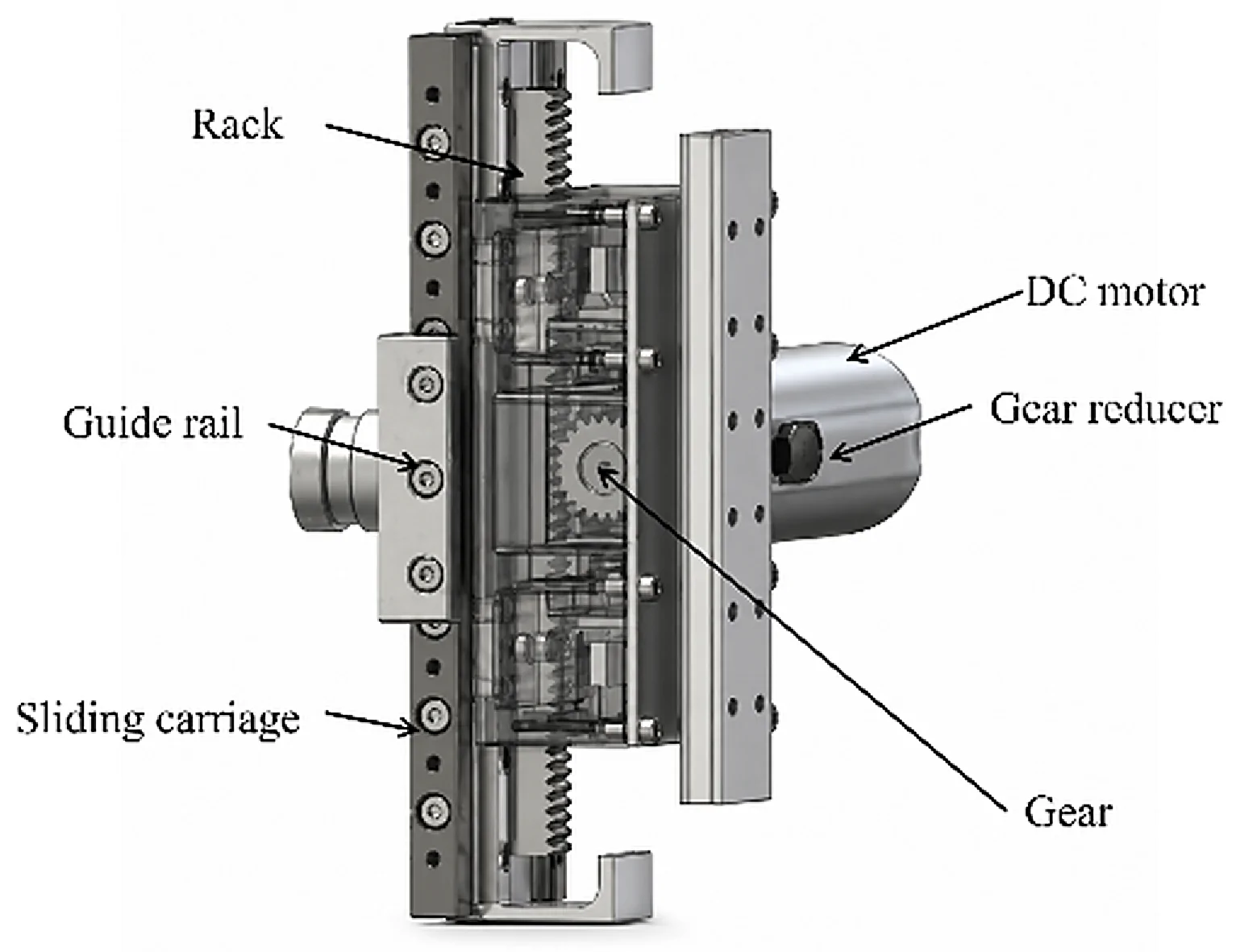
Arc-voltage-control actuator and rack-and-pinion transmission.

The AVC unit uses a DC motor, reduction gear, and rack-and-pinion transmission (Figure 2). Desired velocity is proportional to command voltage and saturates at approximately 20 mm/s. Following established actuator-identification practice [22,23], step-response measurements showed that its velocity dynamics were adequately represented by a first-order lag with time constant $\tau_{m}=0.04$ s:

(1) $$\tau_{m}\dot{v}\left(t\right)+v\left(t\right)=K_{m}u\left(t\right),\;\left|v\left(t\right)\right|\leq 20\;\mathrm{mm}/s.$$

Rack-and-pinion backlash, a principal accuracy limitation in this drive architecture [24], was modeled using a play operator

(2) $$x_{a}\left(k\right)=\left\{\begin{array}{cc} x_{i}\left(k\right)-b/2, & x_{i}\left(k\right)-x_{a}\left(k-1\right)>b/2,\\ x_{i}\left(k\right)+b/2, & x_{i}\left(k\right)-x_{a}\left(k-1\right)<-b/2,\\ x_{a}\left(k-1\right), & \mathrm{otherwise}, \end{array}\right.$$

where $x_{i}$ and $x_{a}$ are ideal and effective displacements, respectively, and the estimated linear backlash is b=0.058 mm. Using the sensor calibration reported in Section 4.1, a working-point form avoids duplicating the intercept in Equation (28):

(3) $$V_{s}\left(t\right)=V_{0}+K_{v}\Delta x_{a}\left(t\right),\;K_{v}=1.56\;V/\mathrm{mm}.$$

The thermal response from the static equivalent voltage $V_{s}$ to the actual voltage $V_{a}$ was represented as a second-order system, consistent with dynamic arc-control modeling [25]:

(4) $$G_{a}\left(s\right)=\frac{V_{a}\left(s\right)}{V_{s}\left(s\right)}=\frac{\omega_{n}^{2}}{s^{2}+2\zeta \omega_{n}s+\omega_{n}^{2}},$$

with damping ratio ζ=0.70 and natural frequency $\omega_{n}=25$ rad/s. These values imply approximately 4.6% theoretical overshoot, a 0.085 s 10–90% rise time, and a 0.23 s 2% settling time, consistent with the actuator time scale.

**Figure 3:**
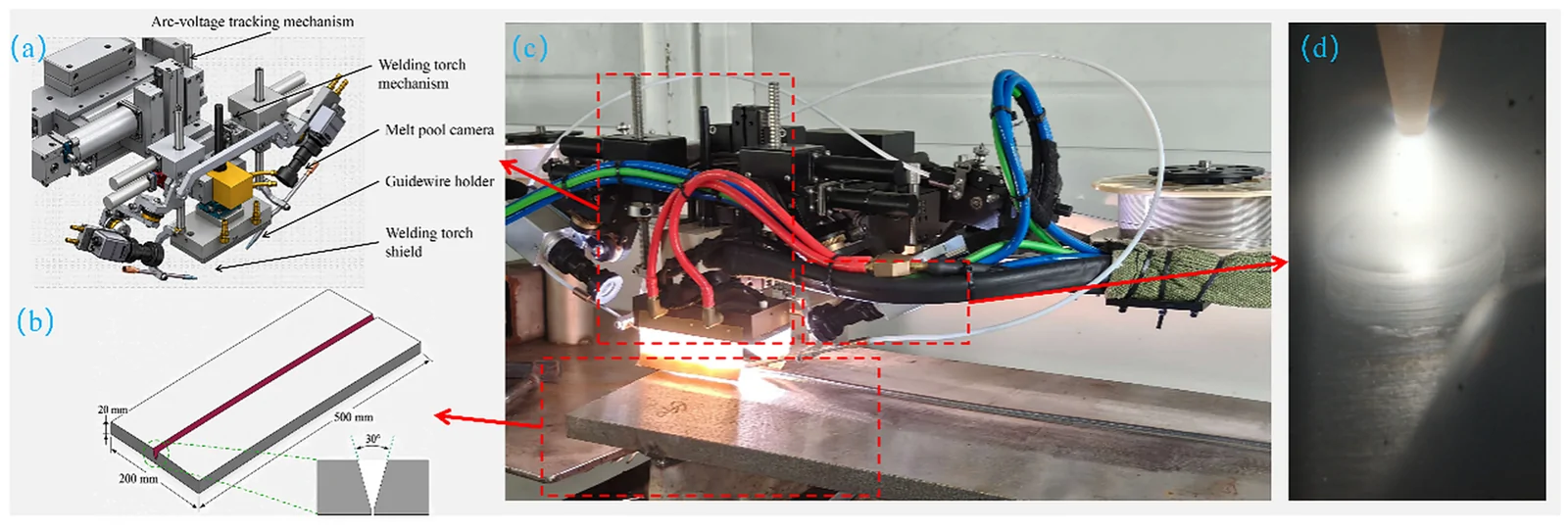
Physical realization of the proposed adaptive AVC scheme: (**a**) integrated narrow-gap TIG welding head and arc-voltage tracking mechanism; (**b**) constrained test-plate groove; (**c**) assembled sensing–actuation welding platform; and (**d**) arc and molten-pool observation.

Figure 3 maps the abstract blocks in Figure 1 to the physical implementation. The arc-voltage tracking mechanism and welding torch in panel (a) realize the actuator and torch-height correction path; the groove geometry in panel (b) defines the constrained process environment; the integrated platform in panel (c) combines voltage acquisition, actuation, wire feeding, and observation; and panel (d) shows the arc–molten-pool region from which disturbance-contaminated voltage feedback originates. This hardware mapping clarifies how reconstructed feedback, effective-error decisions, and adaptive commands close the loop on the real welding system.

#### 3.1.2. Adaptive Kalman Filter Feedback Reconstruction

The measured pulsed voltage was treated as the sum of the control-relevant voltage state and feedback-channel disturbances. A linear Kalman estimator [26] was applied with a state vector containing voltage and its derivative:

(5) $$x_{k}={\left[V_{a,k}\;\;{\dot{V}}_{a,k}\right]}^{T}.$$

After discretizing Equation (4) with sampling period $T_{s}=1$ ms using the second-order Taylor approximation retained in the controller implementation, the model is

(6) $$\begin{array}{cc} x_{k} & =A_{d}x_{k-1}+B_{d}V_{s,k-1}+w_{k-1}, \end{array}$$

(7) $$\begin{array}{cc} y_{k} & =Hx_{k}+v_{k},\;H=\left[1\;0\right], \end{array}$$

where $w_{k-1}∼N\left(0,Q\right)$ represents plant uncertainty and backlash, while $v_{k}∼N\left(0,R\right)$ represents acquisition noise and pulse-related background variation. The initial values of Q and R were determined from the model uncertainty and open-loop voltage fluctuation characteristics, respectively, and were subsequently included as optimization variables in the NSGA-II tuning. The AKF prediction and update follow the standard KF formulation with adaptive measurement covariance:

(8) $$\begin{array}{cc} {\hat{x}}_{k|k-1} & =A_{d}{\hat{x}}_{k-1|k-1}+B_{d}V_{s,k-1}, \end{array}$$

(9) $$\begin{array}{cc} P_{k|k-1} & =A_{d}P_{k-1|k-1}A_{d}^{T}+Q, \end{array}$$

(10) $$\begin{array}{cc} K_{k} & =P_{k|k-1}H^{T}{\left(HP_{k|k-1}H^{T}+R_{k}\right)}^{-1}, \end{array}$$

(11) $$\begin{array}{cc} {\hat{x}}_{k|k} & ={\hat{x}}_{k|k-1}+K_{k}\left(y_{k}-H{\hat{x}}_{k|k-1}\right), \end{array}$$

(12) $$\begin{array}{cc} P_{k|k} & =\left(I-K_{k}H\right)P_{k|k-1}. \end{array}$$

The reconstructed feedback is ${\hat{V}}_{k}=\left[1\;0\right]{\hat{x}}_{k|k}$. The implementation also includes a 0.02 V measurement dead zone and adapts the measurement covariance to reduce the influence of isolated innovations and repeated held samples:

(13) $$R_{k}=\left\{\begin{array}{cc} 10R, & |y_{k}-H{\hat{x}}_{k|k-1}|>0.7\;V,\\ 5R, & |y_{k}-y_{k-1}|<10^{-9}\;V,\\ R, & \mathrm{otherwise}. \end{array}\right.$$

Thus, the AKF fuses the measured voltage with the identified dynamics instead of applying an unstructured moving average. Its measurement confidence changes online through $R_{k}$: a large innovation or held sample temporarily reduces reliance on the measurement, while normal innovations restore the nominal covariance. This constitutes the estimator-adaptation layer of the proposed AVC.

### 3.2. Feedback-Reconstruction-Driven Adaptive Control

#### 3.2.1. Amplitude–Duration–Area Effective-Error Identification

The raw reconstructed-feedback error is

(14) $$e_{k}^{\mathrm{raw}}=V_{\mathrm{ref}}-{\hat{V}}_{k}.$$

Open-loop statistics indicated that most pulse-related adjacent transitions were below 0.40 V. We therefore selected an amplitude boundary $e_{\mathrm{th}}=0.35$ V. While $|e_{k}^{\mathrm{raw}}|>e_{\mathrm{th}}$, the continuous exceedance duration $T_{e}$ and absolute error area $A_{e}$ are accumulated:

(15) $$T_{e}\leftarrow T_{e}+T_{s},\;A_{e}\leftarrow A_{e}+\left|e_{k}^{\mathrm{raw}}\right|T_{s}.$$

Both states reset to zero when the error returns within the boundary. An error is declared effective when

(16) $$|e_{k}^{\mathrm{raw}}|\;>e_{\mathrm{th}}\;\wedge \;\left(T_{e}>T_{\mathrm{th}}\;\vee \;A_{e}>A_{\mathrm{th}}\right),$$

where $e_{\mathrm{th}}$, $T_{\mathrm{th}}$, and $A_{\mathrm{th}}$ denote the amplitude, duration, and area thresholds, respectively. The thresholds were fixed during controller optimization and were not included in the NSGA-II search space. This logic rejects isolated excursions but preserves a moderate error that accumulates over time.

#### 3.2.2. Online Fuzzy-PID Gain Adaptation

The controller receives $e_{k}^{\mathrm{eff}}=e_{k}^{\mathrm{raw}}$ only after this condition is satisfied and otherwise receives zero. The adaptation therefore responds to control-relevant reconstructed deviations rather than to every measured pulse. A two-input Mamdani fuzzy system uses the reconstructed error and its scaled change rate to generate PID-gain corrections [27]. The fuzzy layer is activated after the ignition transient at 0.909 s and updated every 24 ms according to the effective feedback interval. Its error-change input is calculated from successive fuzzy-update errors, scaled by the 1 ms controller interval, and clipped to [−249,249]. Seven linguistic sets (NB, NM, NS, ZO, PS, PM, and PB) are used for each input. The complete 49-rule base retained from the controller implementation is reported in Table 1. Large errors increase proportional action and suppress integral action; near-zero errors with rapid motion reduce proportional action and increase derivative damping. If $f_{j}\left(k\right)$ is the defuzzified output for j∈{p,i,d}, the corrections and gains are

(17) $$\begin{array}{cc} \Delta K_{j}^{\mathrm{raw}}\left(k\right) & =\lambda_{f}s_{j}f_{j}\left(k\right), \end{array}$$

(18) $$\begin{array}{cc} \Delta K_{j}\left(k\right) & =\left(1-\alpha_{f}\right)\Delta K_{j}\left(k-1\right)+\alpha_{f}\Delta K_{j}^{\mathrm{raw}}\left(k\right), \end{array}$$

(19) $$\begin{array}{cc} K_{j}\left(k\right) & =K_{j0}+\Delta K_{j}\left(k\right). \end{array}$$

Here, $K_{j0}$ is the offline-tuned base gain, $\Delta K_{j}\left(k\right)$ is the online adaptive correction, and $s_{j}$ denotes the scaling factor of the *j*th PID-gain correction. Consequently, the control law changes its response magnitude in real time without changing the prescribed target $V_{\mathrm{ref}}$. In this sense, adaptation concerns feedback confidence and controller gains, rather than autonomous target-voltage generation. The integral state and saturated voltage command are

(20) $$\begin{array}{cc} I_{k} & =I_{k-1}+e_{k}^{\mathrm{eff}}T_{s}, \end{array}$$

(21) $$\begin{array}{cc} d_{k} & =d_{k-1}+T_{s}N_{f}\left[-K_{d}\left(k\right)\frac{{\hat{V}}_{k}-{\hat{V}}_{k-1}}{T_{s}}-d_{k-1}\right], \end{array}$$

(22) $$\begin{array}{cc} u_{k}^{*} & =K_{p}\left(k\right)e_{k}^{\mathrm{eff}}+K_{i}\left(k\right)I_{k}+d_{k}, \end{array}$$

(23) $$\begin{array}{cc} u_{k} & =\mathrm{sat}(u_{k}^{*},u_{\min},u_{\max}). \end{array}$$

Hence, the derivative action is calculated from successive reconstructed-voltage samples and low-pass filtered with $N_{f}=30$; it is not differentiated directly from the raw measurement. Before fuzzy adaptation is enabled, the implementation uses error-dependent staged PID gains to manage the ignition transient.

### 3.3. Joint Estimator–Controller Parameter Tuning

Controller tuning was formulated as a constrained bi-objective problem. The decision vector was

(24) $$\theta ={\left[K_{p0},K_{i0},K_{d0},q_{1},q_{2},R,\lambda_{f},\alpha_{f}\right]}^{T},$$

where $q_{1}$ and $q_{2}$ are the diagonal elements of Q. The fuzzy rule base, $K_{v}$, actuator saturation, and error-identification thresholds remained fixed.

Simulations used a 1 ms integration and control step and a 5 s duration. The reference rose from 0 to 12.6 V during ignition and settled at 13.0 V after 0.60 s. A 0.5 V equivalent step disturbance was applied at 2.0 s, followed by a 0.30 V/s ramp during 3.0–5.0 s to emulate slowly varying thermal deformation. Zero-mean Gaussian measurement noise with standard deviation 0.14 V was combined with a positive 0.50 V pulse train having a 20 ms period and 5 ms width. Table 2 lists the remaining reproducibility settings.

The tracking objective was the voltage root-mean-square error (RMSE)

(25) $$J_{V}=\sqrt{\frac{1}{N_{e}}\sum_{k\in \Omega_{e}}{\left(V_{\mathrm{ref},k}-V_{a,k}\right)}^{2}},$$

where $\Omega_{e}=\left\{k:t_{k}\geq 0.60\;s\right\}$ begins after the reference transition. Actuator burden was represented by cumulative effective travel

(26) $$S_{p}=\sum_{k\in \Omega_{e},\;k>1}\left|x_{a}\left(k\right)-x_{a}\left(k-1\right)\right|,$$

and the dimensionless optimization objective was $J_{P}=S_{p}/{\bar{S}}_{p,\mathrm{base}}$, where ${\bar{S}}_{p,\mathrm{base}}$ is the 20-seed baseline mean. Normalization changes the reporting scale but not Pareto dominance. The optimization was

(27) $$\min_{\theta \in \Theta }\left[J_{V}\left(\theta \right),J_{P}\left(\theta \right)\right],$$

Feasible solutions were required to satisfy $r_{\mathrm{sat}}\leq 0.05$, $J_{V}\leq 1.5{\bar{J}}_{V,\mathrm{base}}$, steady-state error ≤0.25 V, maximum absolute error ≤1.0 V, and $S_{p}\leq 2{\bar{S}}_{p,\mathrm{base}}$, with no NaN, Inf, or stick-protection event. NSGA-II used Latin-hypercube initialization to improve coverage [20]. Each candidate was evaluated over five search seeds, and representative solutions were re-evaluated using 20 additional validation seeds.

## 4. Experiments

The experiments evaluated model calibration, parameter optimization, reconstruction performance, and real-machine voltage regulation. First, calibration and open-loop acquisition establish the plant sensitivity and pulse-transition scale used by the model and effective-error rule. Second, Pareto optimization and 20-seed simulation test the joint accuracy–travel objective under short pulses and sustained disturbances. Third, reconstruction analysis tests whether feedback discontinuities are suppressed with delay shorter than the error-persistence threshold. Fourth, the 13.0 V controller comparison and six multi-condition records test whether the complete adaptive loop improves or maintains voltage regulation without hiding its adjustment burden. All reported comparisons retain the prescribed voltage target.

### 4.1. Experimental Equipment and Materials

The base material was a 304 stainless-steel plate measuring 500×200×20 mm, and the filler was 1.0 mm-diameter ER308 wire. The plate had a double-sided V groove with a 30° included angle, a 3 mm root face, and a 1 mm root gap. High-purity argon (99.999%) was delivered through the torch and an external shielding enclosure. The material compositions are listed in Table 3.

**Figure 4:**
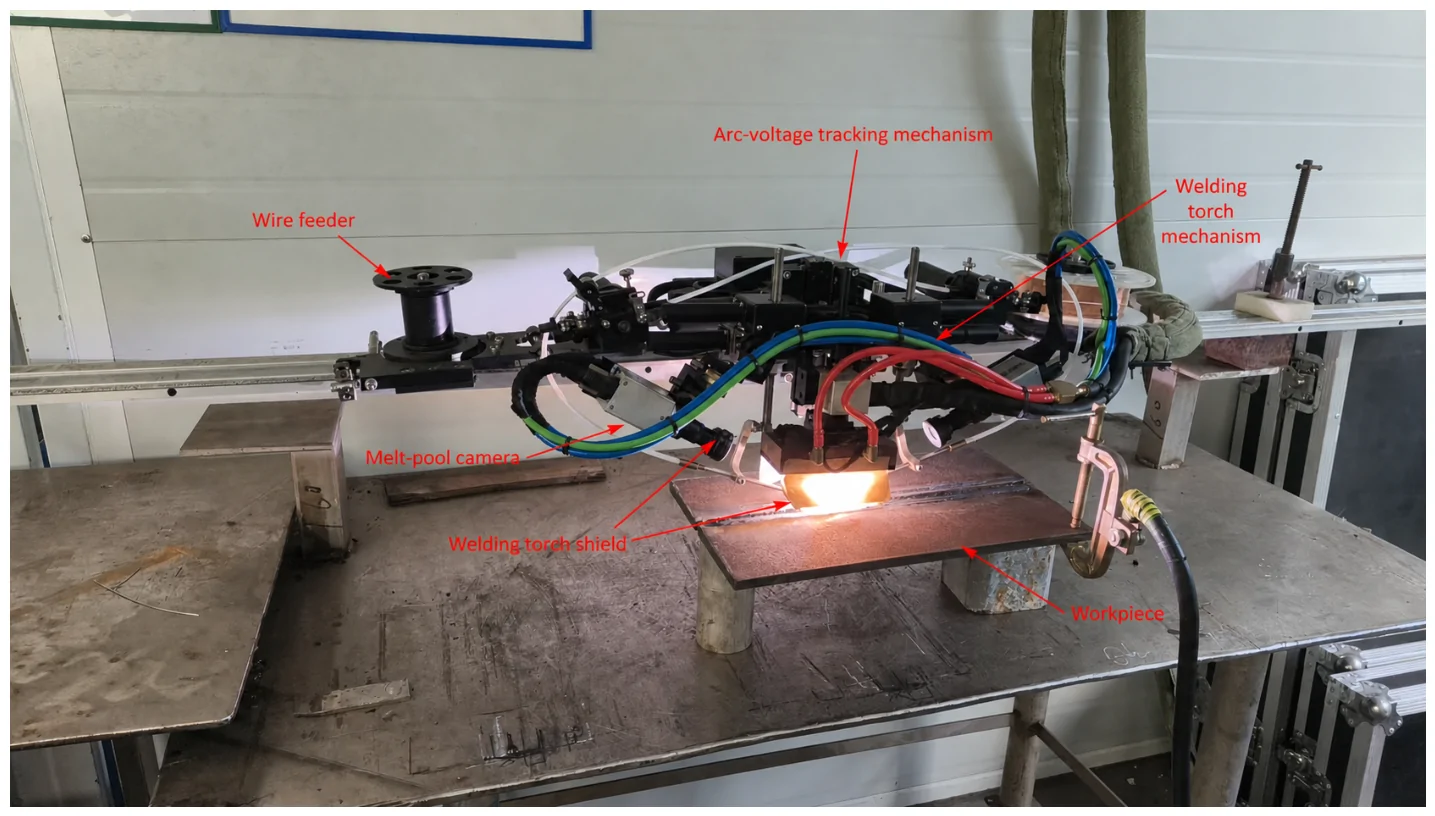
Annotated on-site real-machine welding validation. Red labels identify the wire feeder, arc-voltage tracking mechanism, welding torch mechanism, melt-pool camera, welding torch shield, and workpiece.

The experimental platform comprised a narrow-gap welding head, linear rail, control cabinet, welding power source, and water-cooling system. The head integrated the AVC slide, torch, wire feeder, and molten-pool camera. Figure 4 shows the assembled head operating during an additional real-machine validation run; the correspondence to the proposed sensing–decision–adaptation scheme is presented with the method in Section 3. The Beckhoff controller executed the AVC task at 1 ms, whereas the Scope logger exported peak-voltage, welding-current, AVC-displacement, and controller-status channels every 3 ms. These two time bases are distinguished below: 1 ms is used for controller and simulation updates, and 3 ms is used for analysis of the recorded welding signals. Process parameters are reported in Table 4.

The 13.6 V entry in Table 4 is the measured open-loop operating voltage used for characterization; the closed-loop comparison used a 13.0 V controller reference. This distinction prevents the open-loop characterization level from being interpreted as the commanded welding target.

### 4.2. Arc-Voltage Sensor Calibration and Open-Loop Characterization

#### 4.2.1. Voltage–Displacement Calibration

The peak voltage variation was measured at incremental AVC displacements of 0.4, 0.8, 1.2, and 1.6 mm relative to the initial arc position under otherwise constant welding conditions. Each position was repeated three times (Table 5). Regression was performed on the four position-wise means; the repetitions were used to quantify within-position dispersion rather than treated as 12 independent calibration levels. Linear regression gave

(28) $$V_{m}=10.04+1.56x_{\mathrm{avc}},$$

**Figure 5:**
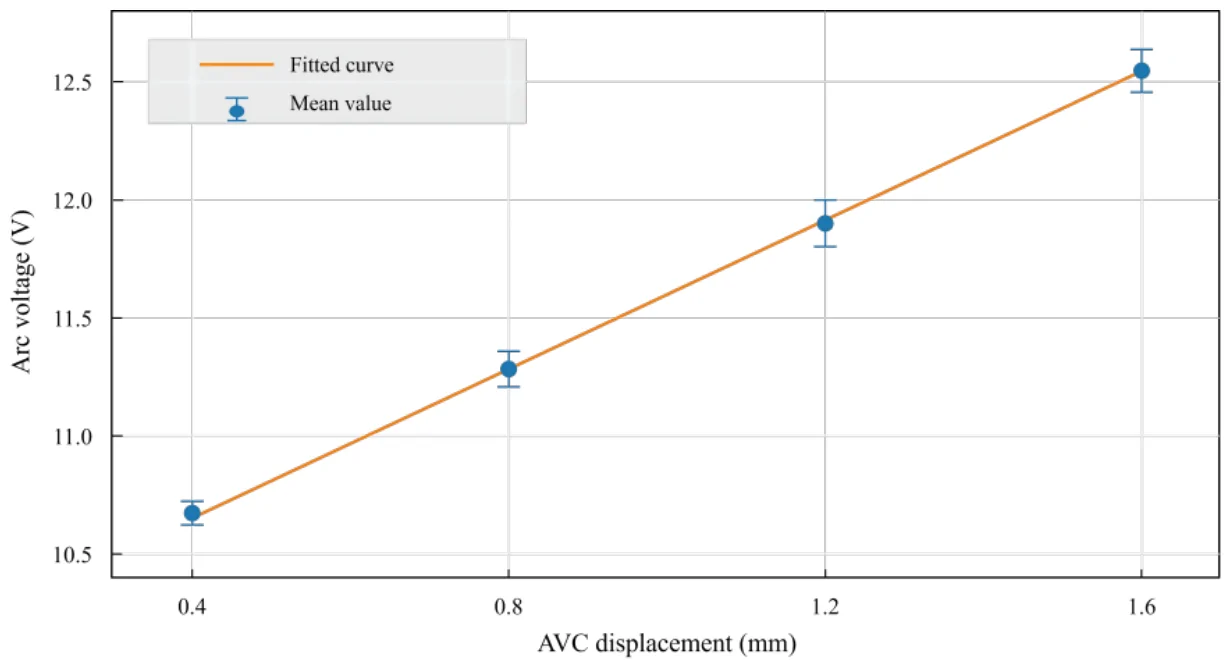
Linear relationship between peak arc voltage and AVC displacement.

The fit had $R^{2}=0.9998$, establishing a voltage–displacement sensitivity $K_{v}=1.56$ V/mm (Figure 5). The regression residual analysis showed an RMSE of 0.0102 V and a maximum residual of 0.013 V based on the four position-wise mean values. According to the calibrated sensitivity, the fitting RMSE corresponds to approximately 0.0065 mm displacement error, indicating that the calibration uncertainty is sufficiently small for the control-oriented voltage–displacement model.

#### 4.2.2. Open-Loop Voltage Acquisition

**Figure 6:**
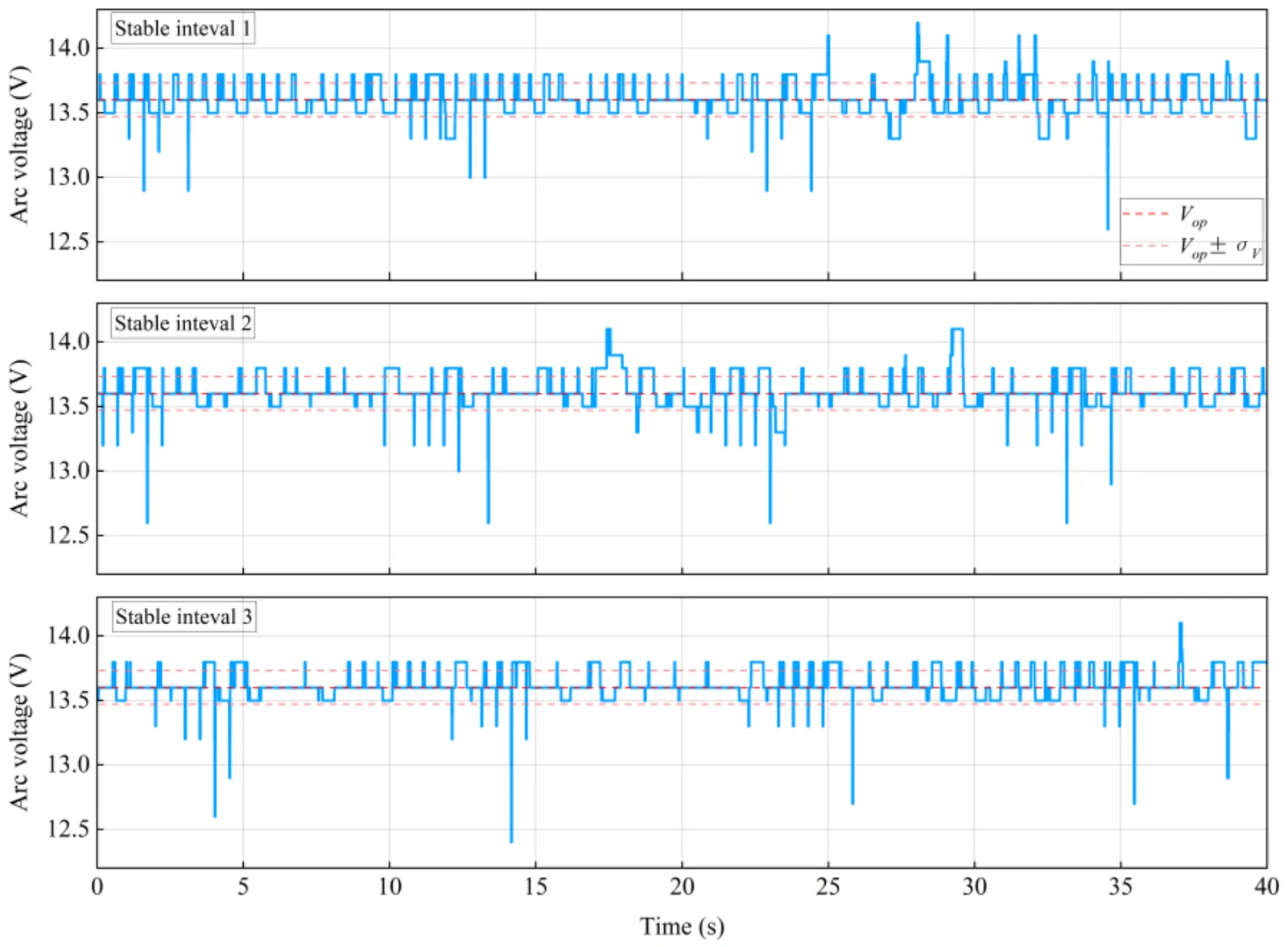
Stable open-loop arc-voltage signals under pulsed TIG welding.

With the AVC actuator held stationary, the Beckhoff control system acquired three stable-welding segments at a 3 ms sampling period over a total duration of 120 s. Similar arc-sensing measurements have been used for transfer monitoring and pulsed-TIG tracking [5,10]. The mean and standard deviation were 13.6 and 0.14 V, respectively (Figure 6). The pulsed signal showed a plateau–step pattern. To characterize its instantaneous transitions, the nonzero adjacent increment was defined as

(29) $$\Delta V_{k}=V_{m,k}-V_{m,k-1}.$$

Among 807 nonzero increments, 92.57% had $|\Delta V_{k}|\;\leq 0.40$ V, whereas only 4.83% exceeded 0.60 V (Table 6). Larger jumps were short-lived rather than persistent voltage drift.

### 4.3. Joint Parameter Optimization and Simulation Validation

#### 4.3.1. Pareto-Front Characteristics

**Figure 7:**
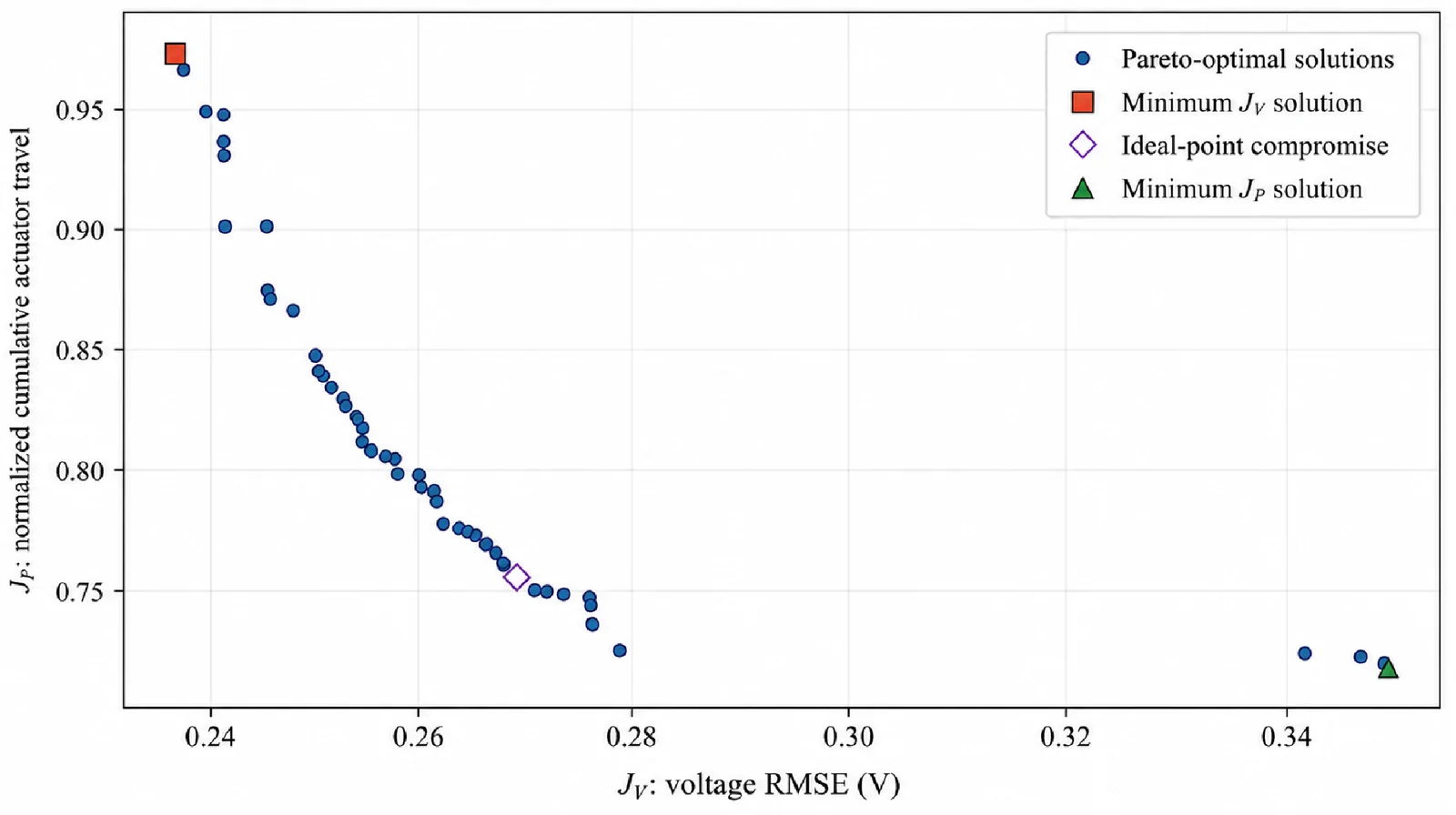
Pareto front and representative solutions in objective space.

NSGA-II used 60 individuals for 40 generations. The stored final front contained 60 entries and 57 unique objective pairs after duplicate objective coordinates were removed (Figure 7). The front demonstrates direct competition between voltage accuracy and cumulative actuator travel. We selected the minimum-$J_{V}$ solution, the minimum-$J_{P}$ solution, and a compromise solution with the smallest normalized Euclidean distance to the ideal point [28]. Table 7 reports voltage RMSE and the corresponding unnormalized travel $S_{p}$ for physical interpretation.

Relative to the baseline, the minimum-$J_{V}$ solution reduced voltage error by 19.4% and travel by 3.9%. The compromise solution further reduced travel but increased voltage error, whereas the minimum-$J_{P}$ solution increased $J_{V}$ by 19.3%. A final choice based only on one Pareto realization would therefore be sensitive to the particular noise sequence.

#### 4.3.2. Random-Seed Validation

Twenty-seed validation quantified RMSE, mean absolute error (MAE), final steady-state error, and ramp-disturbance error (Table 8) [21]. The compromise and minimum-$J_{P}$ parameterizations reduced travel but markedly increased steady-state and ramp errors, showing that aggressive suppression of motion compromises adaptive compensation for persistent deviations. The minimum-$J_{V}$ parameterization gave the lowest RMSE and best ramp tracking while maintaining similar travel to the baseline. Relative to the baseline parameterization, it reduced RMSE, MAE, and ramp error by 15.8%, 22.1%, and 51.0%, respectively, while cumulative travel decreased by 1.9%. Its steady-state error increased by only 0.0039 V. This minimum-$J_{V}$ vector defined the tuned adaptive configuration (Table 9) deployed in the welding experiment.

**Figure 8:**
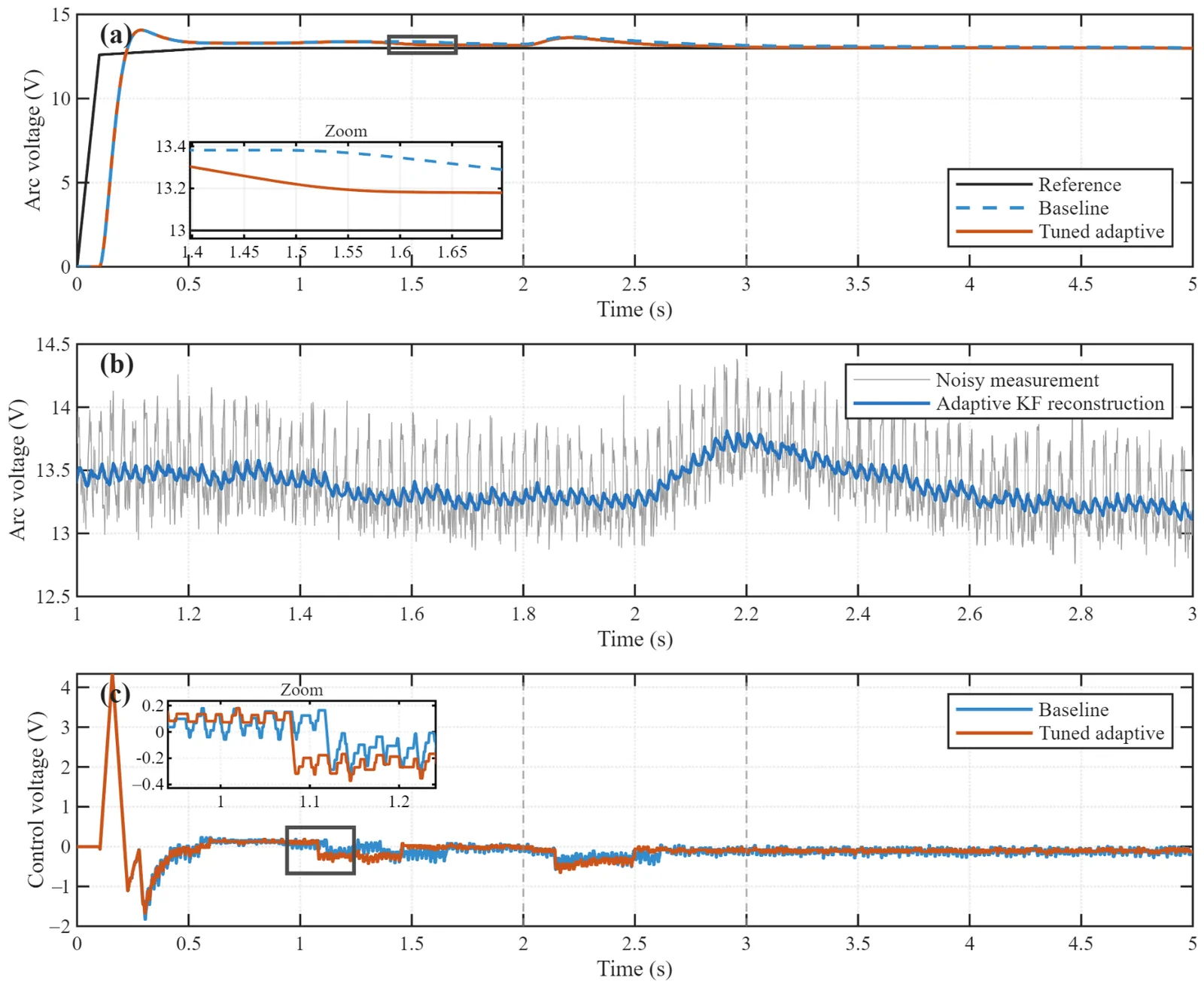
Simulated closed-loop response of the baseline and tuned adaptive configurations: (**a**) arc voltage; (**b**) noisy measurement and AKF reconstruction for the tuned configuration; and (**c**) control voltage. Both configurations retain a prescribed 13.0 V process target.

Figure 8a shows that both parameterizations maintained the prescribed target, but the tuned adaptive response had smaller post-step deviation and followed the ramp more closely. Figure 8b shows that AKF reconstruction attenuated the periodic measurement spikes while preserving the slower disturbance response. Figure 8c shows comparable voltage-command magnitudes but fewer rapid changes with the tuned adaptive configuration. Because cumulative travel remained nearly unchanged, the improvement is consistent with more effective feedback-confidence and gain updates rather than greater actuator activity.

**Figure 9:**
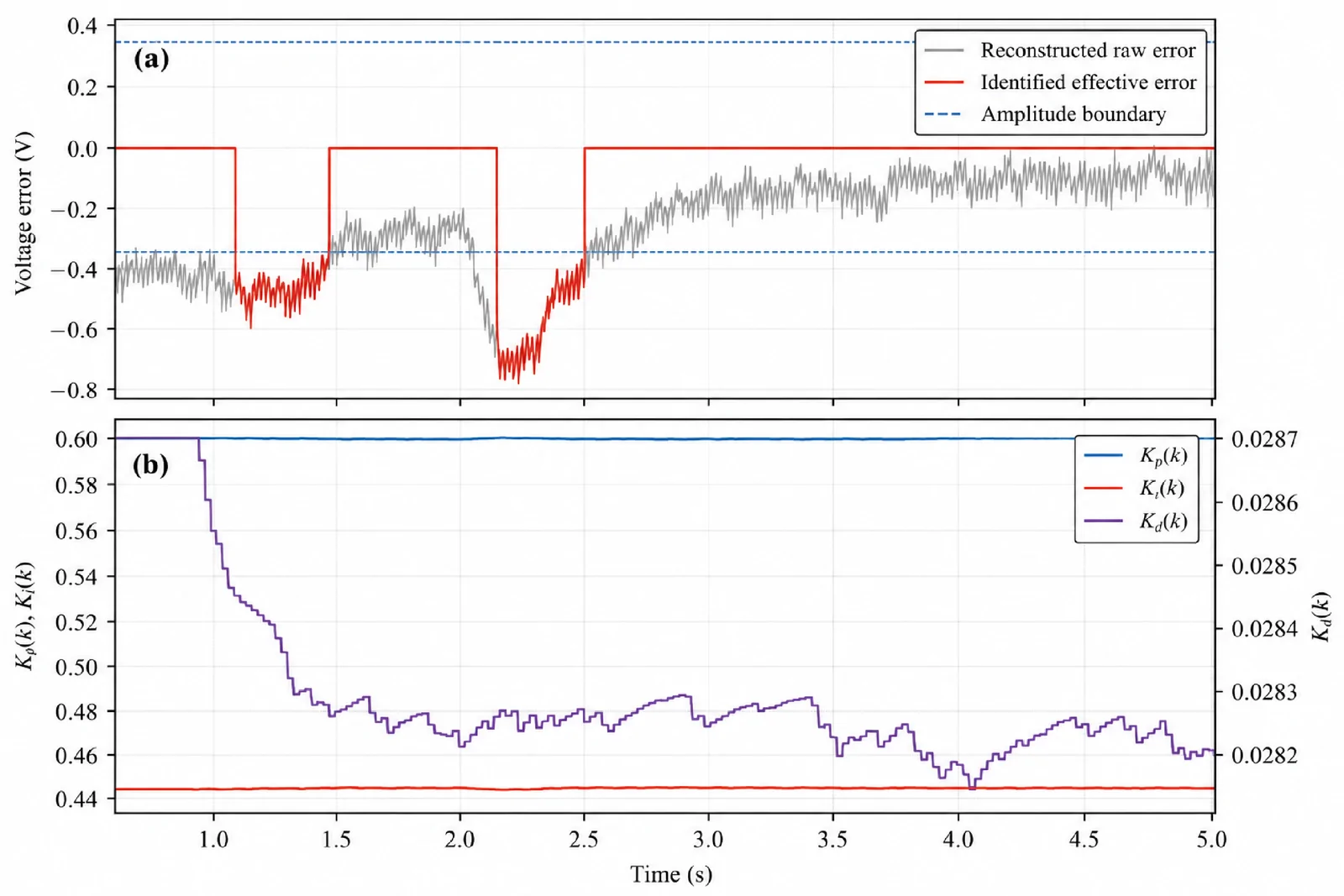
Online decision and adaptation variables for the tuned configuration (validation seed 42): (**a**) reconstructed raw error, identified effective error, and amplitude boundary; and (**b**) time-varying fuzzy-PID gains.

Figure 9 exposes the online variables that distinguish adaptation from offline parameter optimization. The effective-error channel remains zero until the reconstructed deviation satisfies the amplitude–duration–area rule, after which it follows the control-relevant error. For the illustrated validation seed, the online gains varied over $K_{p}=0.598971$–0.600394, $K_{i}=0.441138$–0.442417, and $K_{d}=0.028137$–0.028700. These bounded changes show that the fuzzy layer updates the response gains around the NSGA-II-selected base gains; it does not replace the prescribed reference with a condition-dependent target.

#### 4.3.3. Threshold Sensitivity Analysis

To evaluate the robustness of the effective-error identification thresholds, sensitivity analyses were conducted for the amplitude threshold $e_{\mathrm{th}}$, duration threshold $T_{\mathrm{th}}$, and area threshold $A_{\mathrm{th}}$. During the analysis, the controller parameters, plant model, noise characteristics, and disturbance conditions were kept unchanged. Only one threshold parameter was varied at a time, and the voltage regulation performance and cumulative actuator travel were evaluated using the same 20 validation seeds as the random-seed analysis.

The amplitude threshold $e_{\mathrm{th}}$ was varied from 0.25 to 0.45 V, the duration threshold $T_{\mathrm{th}}$ from 0.05 to 0.15 s, and the area threshold $A_{\mathrm{th}}$ from 0.03 to 0.09 V·s.

(30) $$e_{\mathrm{th}}=0.35\;V,\;T_{\mathrm{th}}=0.10\;s,\;A_{\mathrm{th}}=0.06\;V\cdot s.$$

**Figure 10:**
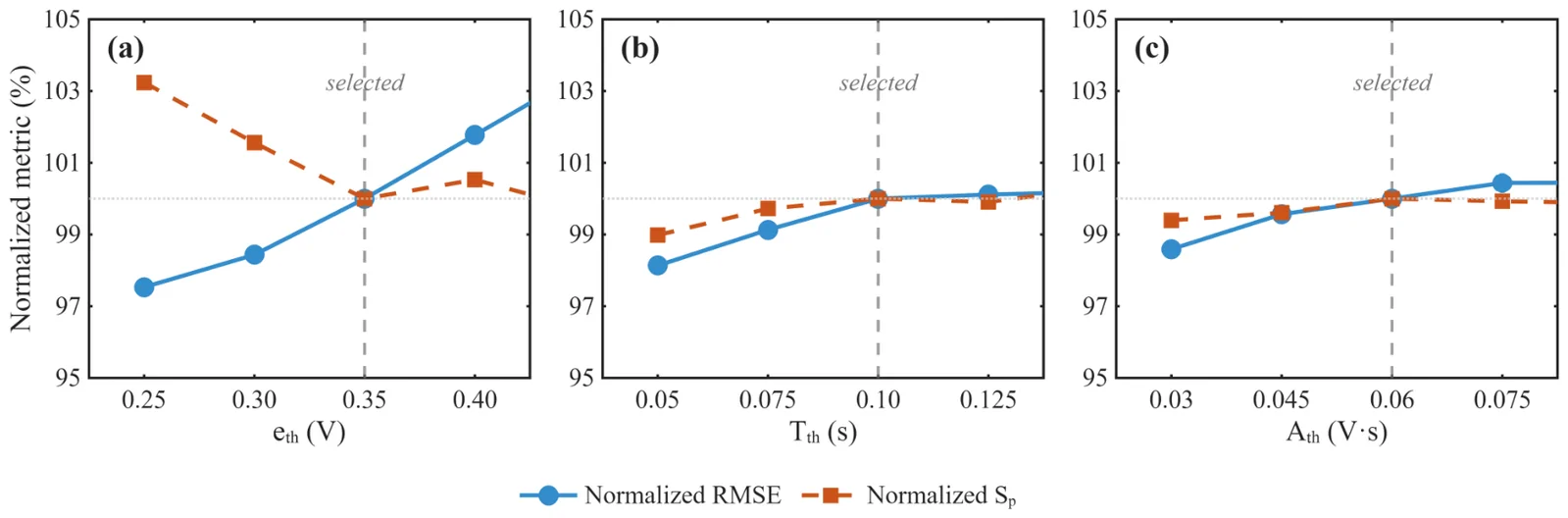
Sensitivity analysis of effective-error identification thresholds: (**a**) amplitude threshold $e_{\mathrm{th}}$, (**b**) duration threshold $T_{\mathrm{th}}$, and (**c**) area threshold $A_{\mathrm{th}}$. The metrics are normalized with respect to the nominal threshold setting.

The normalized RMSE and cumulative actuator travel variations under different threshold settings are shown in Figure 10.

The results show that the proposed AVC framework maintained stable performance within the tested threshold ranges. The amplitude threshold had a relatively larger influence on RMSE because it directly determines whether voltage deviations enter the effective-error identification process. A lower amplitude threshold improves the response to voltage deviations but increases sensitivity to short disturbances. In contrast, variations in the duration and area thresholds resulted in smaller performance changes, indicating that the persistence and accumulated-error criteria mainly regulate the temporal accumulation characteristics of effective errors. No unstable response, abnormal triggering, or saturation was observed within the tested threshold ranges, indicating that the selected thresholds are located in a stable performance region.

#### 4.3.4. Ablation Study

To analyze the contribution of different components in the proposed AVC framework, ablation experiments were conducted. All ablation experiments were conducted under the same simulation framework among the four ablation configurations. The plant model, disturbance conditions, random seeds, and implementation settings were kept unchanged, while a frozen base parameter set was used and only the specified module was removed. Four configurations were evaluated: the full framework, the framework without AKF feedback reconstruction, the framework without effective-error identification (EEI), and the framework without fuzzy-PID gain adaptation (FGA).

**Figure 11:**
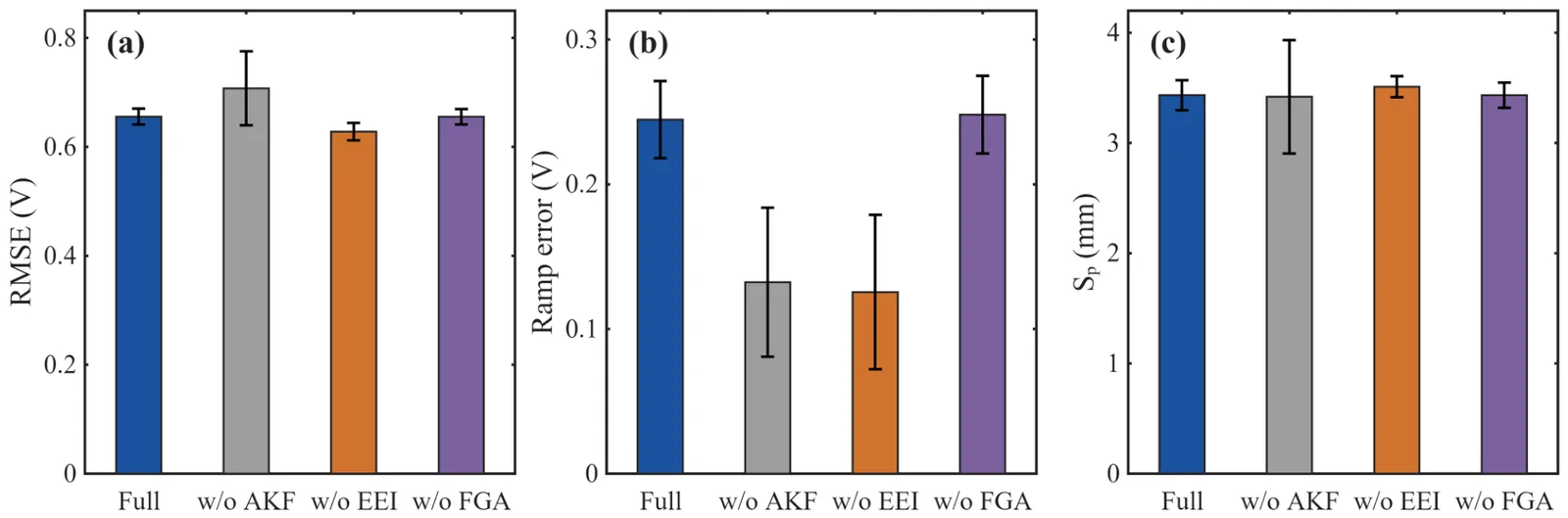
Ablation analysis of the proposed AVC framework: (**a**) voltage RMSE, (**b**) ramp-disturbance error, and (**c**) cumulative actuator travel.

Each configuration was evaluated using 20 independent validation seeds. The voltage RMSE, ramp-disturbance error, steady-state error, cumulative actuator travel, and regulation events were calculated, and the results are summarized in Table 10. The comparisons of RMSE, ramp error, and cumulative actuator travel are shown in Figure 11.

The ablation results show that the complete framework achieved a balanced performance between voltage regulation accuracy and actuator activity. Removing AKF increased the RMSE from 0.6555 V to 0.7073 V and increased the regulation events from 4.25 to 13.30, indicating that feedback reconstruction reduces the influence of measurement noise on control execution.

After removing EEI, the RMSE decreased to 0.6280 V, while the cumulative actuator travel increased from 3.434 mm to 3.510 mm and the regulation events increased from 4.25 to 5.30. This result indicates that EEI affects the balance between error response and actuator activity by identifying control-relevant deviations before execution.

For the fuzzy-PID gain adaptation module, its independent effect was further analyzed. While keeping the AKF, effective-error identification, and base PID parameters unchanged, the fuzzy-PID gain adaptation was disabled and the controller operated with fixed base gains. The results show that the RMSE and cumulative actuator travel remained nearly unchanged after disabling fuzzy-PID gain adaptation. Under the tested conditions, fuzzy-PID gain adaptation did not produce a significant change in the overall control performance. After AKF feedback reconstruction and effective-error identification, the feedback signal provided to the controller already possesses high control relevance. Short-term pulse disturbances and non-geometric voltage fluctuations are effectively suppressed, allowing the base PID controller to accomplish the primary regulation task. Therefore, the performance improvement of the proposed system is mainly attributed to reliable arc-voltage feedback reconstruction and accurate identification of control-relevant errors rather than relying solely on a more complex gain adjustment strategy.

### 4.4. Feedback-Reconstruction Validation

**Figure 12:**
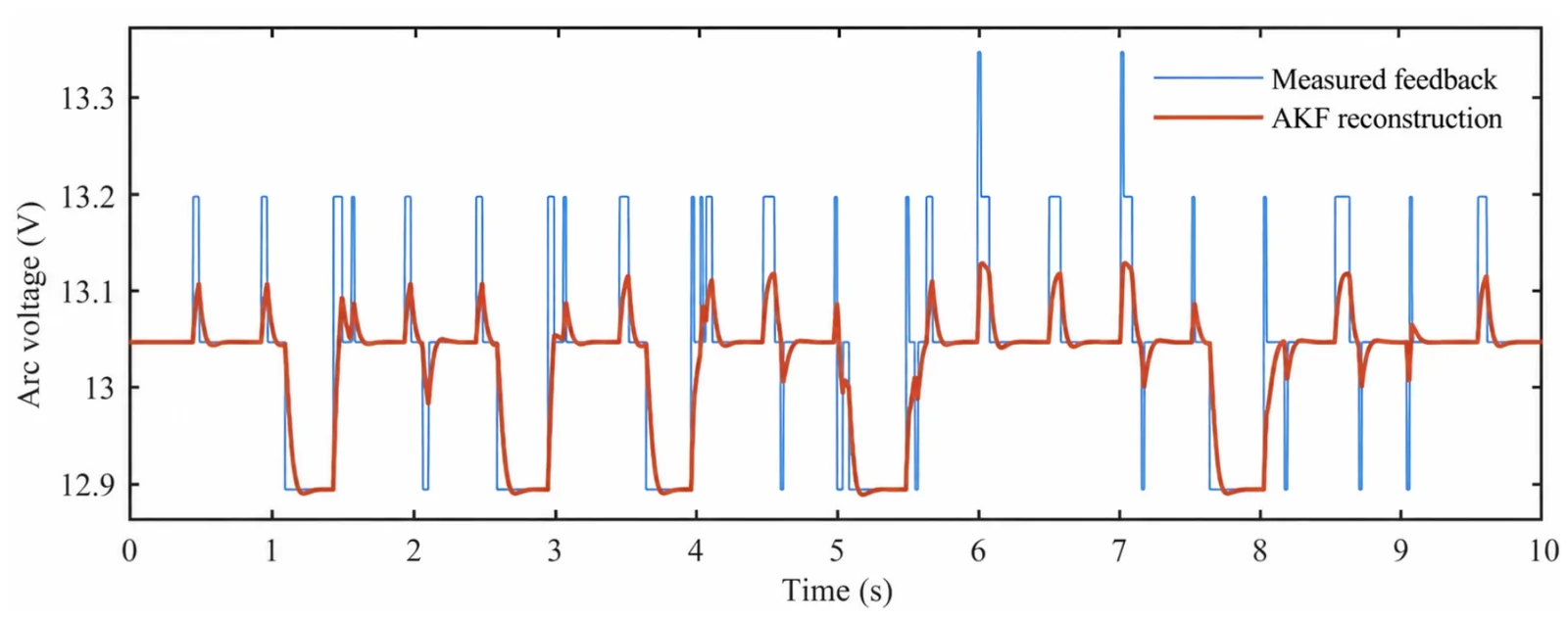
Measured arc-voltage feedback and AKF reconstruction with the tuned adaptive AVC configuration.

Figure 12 tests the AKF reconstruction layer identified in the Abstract and Method using 10 s of stable welding with the tuned adaptive configuration. The measured feedback contains pronounced adjacent-sample jumps, whereas the reconstructed trajectory remains continuous while retaining the slower voltage evolution. The 99th percentile of $|\Delta V_{k}|$ decreased from 0.1495 to 0.0114 V. This reduction is important for the following decision layer because a large measurement increment can otherwise initiate an unnecessary control event.

The reconstruction did not materially alter the operating point: its mean differed from that of the measurement by only 0.01 V, and their trend correlation coefficient was 0.918. After mean removal, normalized cross-correlation estimated a peak lag of six logged samples, or 18 ms [29]. This lag is substantially shorter than the 0.10 s persistence threshold in Equation (16). The result directly supports the intended interface between reconstruction and decision: pulse-scale discontinuities are attenuated without erasing the slower voltage evolution evaluated by the effective-error rule.

### 4.5. Welding Validation

**Figure 13:**
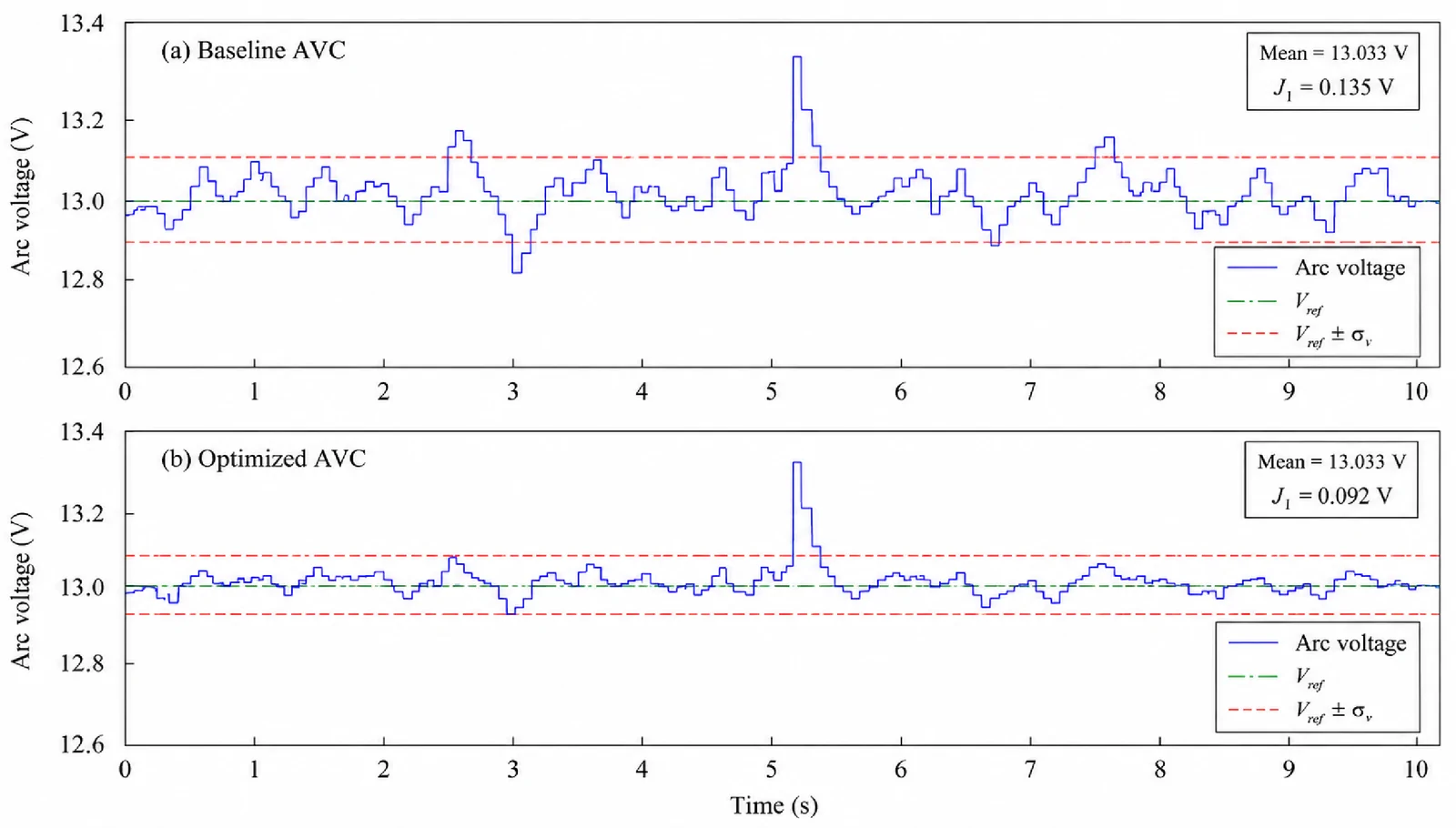
Representative peak arc-voltage signals: (**a**) baseline configuration and (**b**) tuned adaptive configuration.

The baseline-configuration and tuned adaptive-configuration AVC trials used identical process parameters, equipment conditions, and acquisition settings with a prescribed 13.0 V target. The tuned adaptive configuration used the minimum-$J_{V}$ parameter set in Table 9. Three independent welding trials were conducted for each AVC configuration. After stable welding was reached, one continuous 10 s interval was extracted from each trial for analysis. All intervals contained peak-voltage samples recorded every 3 ms, and identical selection criteria and metric definitions were applied. Representative voltage responses from each configuration are shown in Figure 13, and the statistics from the three repeated trials are summarized in Table 11.

For a segment containing *n* samples, the reported quantities were

(31) $$\begin{array}{cc} \bar{V}=\frac{1}{n}\sum_{k=1}^{n}V_{k}, & \;\;\;\;\;\;\;\;\;\;s_{V}=\sqrt{\frac{1}{n-1}\sum_{k=1}^{n}{\left(V_{k}-\bar{V}\right)}^{2}}, \end{array}$$

(32) $$\begin{array}{cc} \mathrm{RMSE}=\sqrt{\frac{1}{n}\sum_{k=1}^{n}{\left(V_{k}-13.0\right)}^{2}}, & \;\;\;\;P_{0.25}=\frac{100}{n}\sum_{k=1}^{n}I(|V_{k}-13.0\left|\;\leq 0.25\right). \end{array}$$

An AVC adjustment event was counted once at each inactive-to-active transition of the recorded regulation-status signal; consecutive active samples therefore represented one adjustment event rather than multiple adjustments. The voltage regulation metrics and adjustment events from the three independent welding trials were statistically summarized for each AVC configuration.

Both configurations maintained a mean voltage close to 13.03 V, indicating that parameter tuning did not shift the operating point. Across the three independent welding trials, the tuned adaptive configuration achieved lower voltage fluctuation than the baseline configuration. The mean standard deviation across the three records decreased from 0.1349 V to 0.1109 V, and the RMSE decreased from 0.1375 V to 0.1104 V. The fraction of samples within ±0.25 V increased from 92.14% to 96.72%.

Meanwhile, the average number of adjustment events decreased from 48.33 for the baseline configuration to 44.33 for the tuned adaptive configuration. These results indicate that the proposed method reduced arc-voltage fluctuation without increasing actuator adjustment activity.

### 4.6. Multi-Condition Real-Machine Validation

Additional real-machine tests examined whether the control architecture retained stable behavior under different prescribed AVC reference voltages. Three target levels, 11.4, 12.2, and 13.2 V, were tested twice, producing six separately recorded stable-welding segments. The target was prescribed before each run; these tests evaluate online adaptation around different operating levels and do not imply autonomous target-voltage generation. Each record contained time, arc voltage, welding current, and AVC displacement at 3 ms intervals. A representative 30 s steady-state welding segment was extracted from each record after excluding arc ignition and transient adjustment periods. Each interval contained 10,000 samples collected at 3 ms intervals. Mean welding current ranged from 191.18 to 194.23 A, indicating that the pulsed-current program remained comparable across the six records.

The voltage statistics followed the definitions above, with the reference voltage replaced by the prescribed target for each run. Because the additional log did not contain the binary regulation-status channel used in Table 11, actuator burden was evaluated directly from the recorded displacement. To normalize actuator travel with respect to the stable-welding duration $T_{w}$:

(33) $$\nu_{p}=\frac{S_{p}}{T_{w}}=\frac{1}{T_{w}}\sum_{k=2}^{n}\left|x_{a}\left(k\right)-x_{a}\left(k-1\right)\right|.$$

This measure is the physical-record counterpart of the displacement objective used in the simulation rather than an inferred count of controller events.

**Figure 14:**
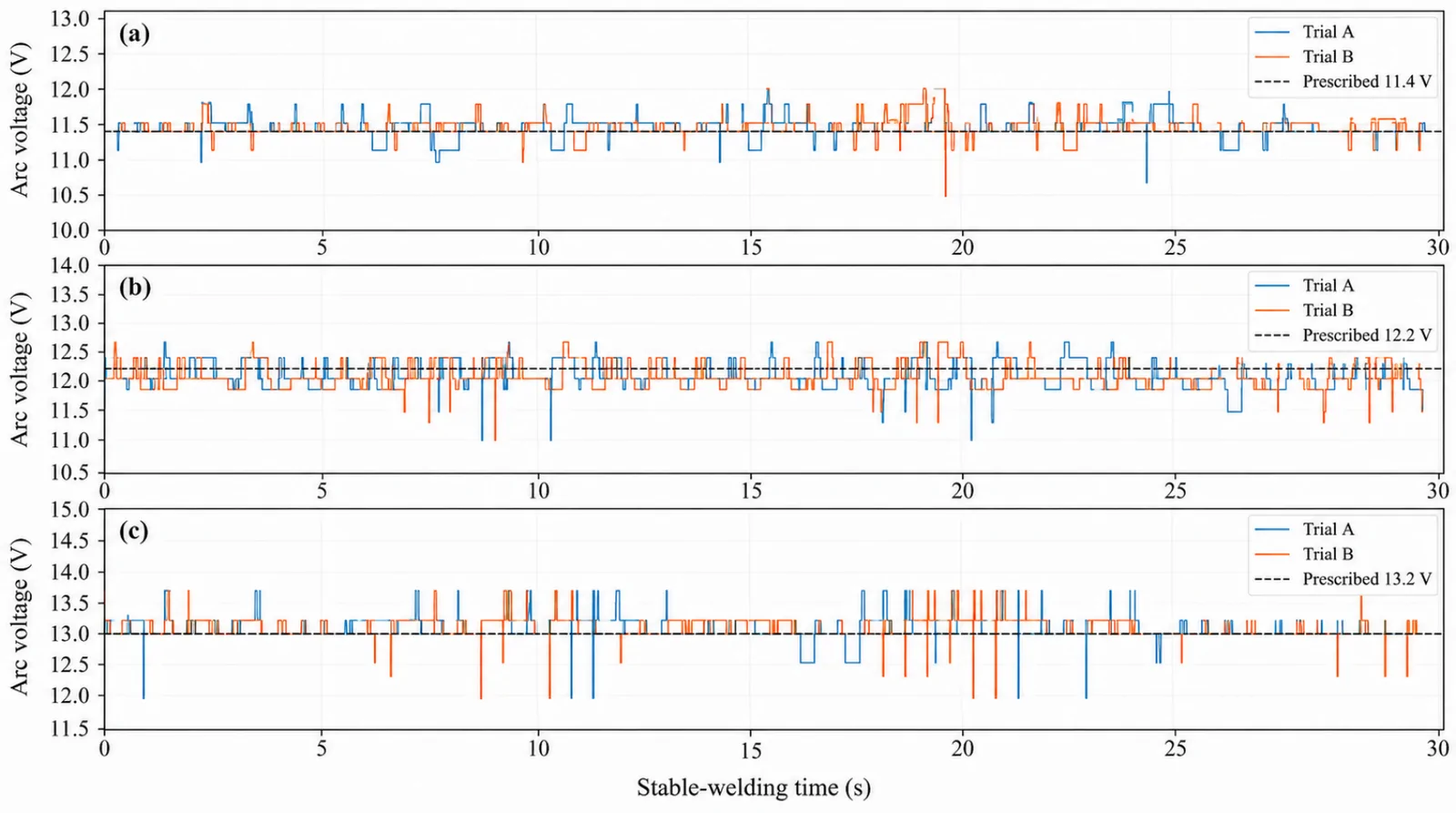
Six additional real-machine 30 s steady-state records at prescribed AVC targets of (**a**) 11.4 V, (**b**) 12.2 V, and (**c**) 13.2 V. Trial A and Trial B are separate records extracted after excluding arc ignition and transient adjustment periods.

**Figure 15:**
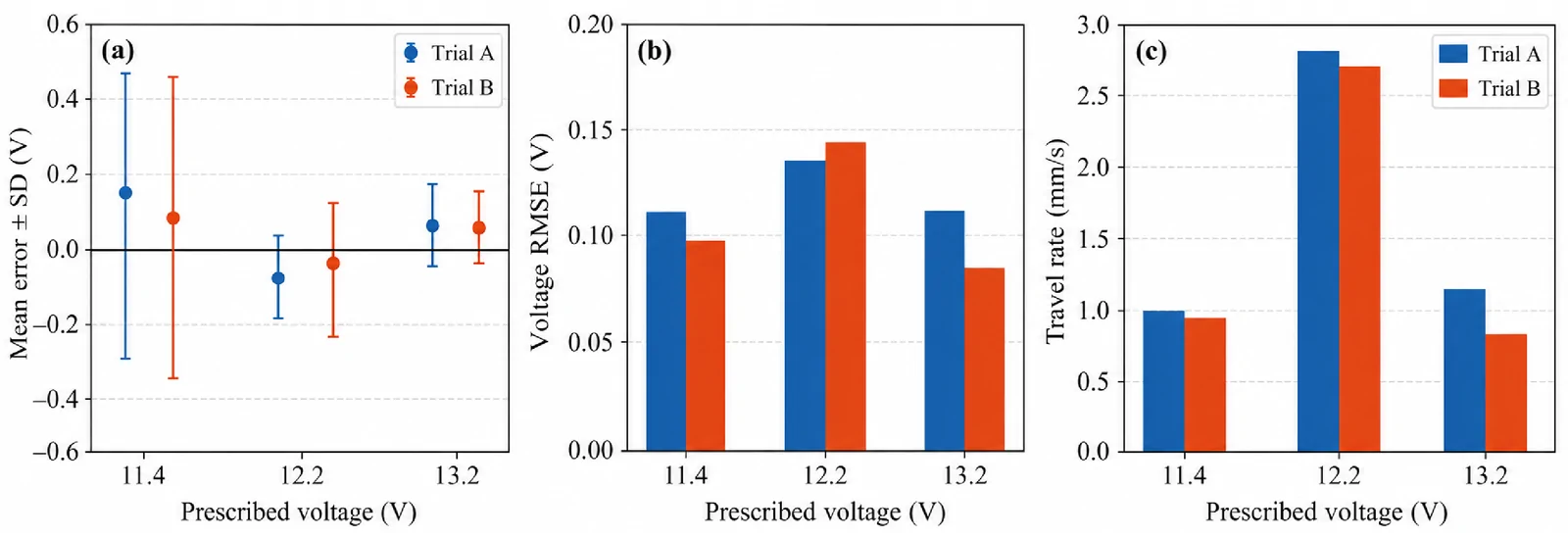
Summary of the multi-condition real-machine tests: (**a**) mean tracking error with sample standard deviation, (**b**) voltage RMSE, and (**c**) recorded actuator-travel rate.

The difference between repeated mean voltages was at most 0.014 V within the selected 30 s intervals, indicating consistent mean voltage regulation across the tested records. However, the regulation performance varied among the prescribed conditions (Figure 14 and Figure 15). The 12.2 V condition achieved the highest $P_{0.25}$ values (98.38–99.02%; Table 12) but required the largest actuator travel rate (2.830–2.892 mm/s). In contrast, the 13.2 V condition reduced actuator travel (0.811–1.175 mm/s) while maintaining comparable voltage regulation performance. The 11.4 V condition showed larger deviations from the prescribed voltage level despite comparable RMSE values.

The six records broaden the physical evidence beyond the 13.0 V controller comparison, but they remain two repetitions per target rather than a factorial study of current, travel speed, groove position, and target voltage. Accordingly, the results demonstrate real-time regulation at three prescribed voltage levels and expose operating-condition sensitivity; they do not yet establish autonomous selection of the target or statistical invariance across all welding procedures.

## 5. Discussion

The simulation, controller comparison, and multi-condition welding records support the proposed adaptive AVC framework from complementary perspectives. In simulation, the tuned adaptive configuration reduced voltage error under step, ramp, pulse, and random disturbances while maintaining actuator travel close to the baseline configuration. In the 13.0 V welding comparison, the proposed method preserved the prescribed operating point, reduced voltage fluctuation, and decreased adjustment events. The additional real-machine records under different voltage targets further verified the stable regulation capability of the proposed method. These results indicate that the performance improvement is not only attributed to controller parameter adjustment but is also closely related to improved feedback quality and control-relevance identification before actuator execution.

The response mechanism can be understood from three aspects: feedback reconstruction, effective-error identification, and adaptive response adjustment. AKF updates the feedback state by combining model prediction and measurement information, transforming the voltage signal affected by pulse switching and process disturbances into a more control-relevant state representation rather than simply smoothing the signal. Subsequently, the amplitude–duration–area criterion evaluates whether a voltage deviation has sufficient control relevance before actuator execution, thereby preventing transient fluctuations from directly triggering unnecessary corrections. The ablation results further show that, after feedback reconstruction and effective-error identification, the feedback signal provided to the controller already possesses high control relevance, and fuzzy-PID gain adaptation mainly provides auxiliary response optimization.

Compared with waveform-synchronized sampling and adaptive filtering approaches, the proposed method introduces an explicit control-relevance identification layer between feedback estimation and control execution. Conventional approaches mainly improve voltage measurement stability by reducing pulse-related fluctuations or selecting stable waveform regions, whereas the proposed framework further determines whether a detected deviation should be converted into actuator correction. Compared with general event-triggered control methods, the proposed decision criterion is established according to pulsed TIG arc-voltage characteristics and experimental calibration rather than general state variables. Therefore, this work focuses on establishing the relationship among feedback reconstruction, error identification, and adaptive control, rather than ranking controllers across different experimental platforms.

Several limitations remain in the present study. The equivalent model mainly describes the dominant dynamic characteristics between actuator motion and arc-voltage response, while factors such as wire feeding, molten-pool behavior, and weld geometry evolution are not explicitly considered. In addition, the effective-error identification thresholds are derived from the current equipment and pulse waveform characteristics, and their applicability requires further evaluation when the current program, electrode configuration, or acquisition settings are changed. The proposed framework also relies on control-oriented assumptions, including local voltage–displacement linearity within the calibrated range and the capability of a low-order model to describe dominant dynamic characteristics. These assumptions have been examined through calibration results, feedback reconstruction analysis, and threshold sensitivity analysis.

## 6. Conclusions

An adaptive arc-voltage controller combining AKF feedback reconstruction, error identification based on amplitude, duration, and area, online fuzzy PID gain adjustment, and NSGA-II base-parameter tuning was developed for narrow-gap pulsed TIG welding. The main findings are as follows:

1. Peak arc voltage varied linearly with AVC displacement over the tested range (sensitivity: 1.56 V/mm). In open-loop data, 92.57% of nonzero adjacent increments were no greater than 0.40 V, providing an empirical basis for separating brief pulse-induced transitions from persistent error.
2. AKF reconstruction reduced adjacent-sample discontinuities while introducing an estimated lag of only 18 ms. The error identifier then combined amplitude, persistence, and accumulated area to allow control action to be driven by persistent reconstructed deviations rather than every voltage fluctuation.
3. Across 20 simulation seeds, the tuned adaptive configuration reduced voltage RMSE by 15.8% and ramp-disturbance error by 51.0%, while cumulative actuator travel decreased by 1.9% relative to the baseline parameterization.
4. The ablation study revealed the roles of individual components in the proposed framework. AKF improved feedback signal stability, effective-error identification selected control-relevant deviations, and fuzzy-PID gain adaptation provided auxiliary response optimization. These results indicate that the main advantage of the proposed method lies in improving feedback information quality and control-relevant error identification rather than solely increasing controller complexity.
5. In welding trials, tuned adaptive AVC preserved the 13.03-V mean operating point, reduced RMSE from 0.1375 V to 0.1104 V, and decreased the number of adjustments from 48.33 to 44.33. Across six additional real-machine records at prescribed targets of 11.4, 12.2, and 13.2 V, the controller maintained stable mean voltage regulation, with repeated means differing by at most 0.014 V.

Future work will combine molten-pool vision and torch-pose information with position-dependent model identification, online bias estimation, and condition-dependent reference generation for all-position welding. In addition, metallographic characterization and mechanical-property evaluation will be conducted to further establish the relationship between arc-voltage regulation performance and final weld quality.

## Figures and Tables

**Table 1:** Fuzzy rules for $\left(\Delta K_{p},\Delta K_{i},\Delta K_{d}\right)$; rows denote reconstructed error and columns denote the scaled error-change input.

	NB	NM	NS	ZO	PS	PM	PB
NB	PB,NB,PS	PB,NB,ZO	PM,NM,NS	PM,NM,NS	PS,NS,NM	ZO,ZO,ZO	ZO,ZO,PS
NM	PB,NB,PS	PM,NM,ZO	PM,NM,NS	PS,NS,NM	ZO,ZO,NS	ZO,ZO,ZO	NS,PS,PS
NS	PM,NM,ZO	PM,NM,ZO	PS,NS,NS	ZO,ZO,NM	ZO,PS,NS	NS,PS,ZO	NS,PM,ZO
ZO	PS,NS,ZO	PS,NS,NS	ZO,ZO,NS	ZO,ZO,NM	NS,PS,NS	NS,PM,NS	NM,PM,ZO
PS	ZO,ZO,ZO	ZO,ZO,NS	NS,PS,NM	NS,PM,NM	NM,PM,NM	NM,PB,NS	NB,PB,ZO
PM	ZO,ZO,ZO	NS,PS,NS	NS,PS,NS	NM,PM,NM	NM,PM,NS	NB,PB,ZO	NB,PB,ZO
PB	NS,PS,ZO	NS,PS,ZO	NM,PM,NS	NM,PM,NS	NB,PB,NS	NB,PB,ZO	NB,PB,PS

**Table 2:** Simulation, controller, and optimization protocol.

Category	Value	Definition
Time base	1 ms; 5 s	Control/integration step; total simulated duration
Actuator command	±10 V; 80 V/s	Command saturation; rate limit
Control dead zone	0.05 V	Minimum command change retained by the controller
Fuzzy scheduling	0.909 s; 24 ms	Enable time; gain-update interval
NSGA-II	60; 40; 0.85	Population; generations; crossover probability
Mutation	1/8; $\eta_{c}=15$; $\eta_{m}=20$	Per-variable probability; SBX and mutation indices
Search seeds	42, 43, 44, 101, 102	Noise realizations used in candidate objectives
Validation seeds	42–44, 101–107, 201–210	Twenty independent re-evaluation seeds

**Table 3:** Chemical composition of the base metal and filler wire (wt.%).

Material	C	Si	Mn	P	S	Cr	Ni	Fe
304	0.04–0.08	0.30–0.60	1.00–1.50	0.025–0.040	0.015–0.025	18.00–19.00	8.00–9.50	Bal.
ER308	0.04–0.08	0.30–0.50	1.50–2.00	0.020–0.030	0.010–0.020	19.50–21.00	9.50–10.50	Bal.

**Table 4:** Pulsed TIG welding parameters.

Current (A)		Voltage (V)	Wire Feed (cm/min)		Travel Speed (mm/min)	Ar Flow (L/min)	
Peak	Base		Peak	Base		Torch	Outer
245	125	13.6	130	125	110	25	50

**Table 5:** Arc-voltage–displacement calibration.

Displacement (mm)	$V_{1}$ (V)	$V_{2}$ (V)	$V_{3}$ (V)	Mean (V)	SD (V)
0.4	10.68	10.62	10.72	10.67	0.05
0.8	11.25	11.23	11.37	11.28	0.08
1.2	11.87	11.82	12.01	11.90	0.10
1.6	12.51	12.48	12.65	12.55	0.09

**Table 6:** Cumulative distribution of nonzero adjacent voltage increments.

Criterion	Count	Cumulative Proportion (%)
$0.10\leq |\Delta V_{k}|\;\leq 0.20$ V	631	78.19
$|\Delta V_{k}|\;\leq 0.30$ V	713	88.35
$|\Delta V_{k}|\;\leq 0.40$ V	747	92.57
$|\Delta V_{k}|\;\leq 0.50$ V	757	93.80
$|\Delta V_{k}|\;\leq 0.60$ V	768	95.17
$|\Delta V_{k}|>0.60$ V	39	4.83
Total	807	100.00

**Table 7:** Objectives for the baseline configuration and representative adaptive-controller parameterizations.

Parameter Set	$J_{V}$ (V)	$S_{p}$ (mm)
Baseline configuration	0.29	1.04
Minimum $J_{V}$	0.24	1.00
Ideal-point compromise	0.27	0.77
Minimum $J_{P}$	0.35	0.74

**Table 8:** Twenty-seed validation (mean ± standard deviation).

Set	RMSE (V)	$S_{p}$ (mm)	MAE (V)	Steady Error (V)	Ramp Error (V)
Baseline configuration	0.2922±0.0063	1.0286±0.0316	0.2297±0.0064	0.0400±0.0248	0.0645±0.0154
Minimum $J_{V}$	0.2459±0.0104	1.0095±0.0292	0.1790±0.0115	0.0439±0.0380	0.0316±0.0154
Compromise	0.2699±0.0107	0.8085±0.0400	0.2347±0.0153	0.1908±0.0508	0.1441±0.0350
Minimum $J_{P}$	0.3483±0.0173	0.7531±0.0528	0.3200±0.0208	0.2487±0.0672	0.2356±0.0429

**Table 9:** Baseline and tuned adaptive-controller parameters.

Set	$K_{p0}$	$K_{i0}$	$K_{d0}$	$q_{1}$	$q_{2}$	*R*	$\lambda_{f}$	$\alpha_{f}$
Baseline configuration	0.3510	0.3116	0.0325	0.0043	0.2500	0.3045	1.4910	0.0540
Tuned adaptive	0.5999	0.4414	0.0287	0.0013	0.5976	0.3221	1.0356	0.0974

**Table 10:** Ablation results of different AVC components.

Method	RMSE (V)	Ramp Error (V)	SSE (V)	$S_{p}$ (mm)	RegEvents
Full	0.6555	0.2447	0.2201	3.434	4.25
w/o AKF	0.7073	0.1323	0.1137	3.419	13.30
w/o EEI	0.6280	0.1255	0.0978	3.510	5.30
w/o FGA	0.6554	0.2481	0.2143	3.433	4.70

**Table 11:** Peak arc-voltage statistics under the two AVC conditions.

Condition	Trials	Mean (V)	SD (V)	RMSE (V)	|e| ≤0.25 (%)	Adjustments
Baseline configuration	3	13.03	0.1349	0.1375	92.14	48.33
Tuned adaptive configuration	3	13.02	0.1109	0.1104	96.72	44.33

**Table 12:** Multi-condition real-machine results calculated from the selected 30 s steady-state intervals.

$V_{\mathrm{ref}}$ (V)	Trial	*n*	$T_{w}$ (s)	$\bar{V}$ (V)	SD (V)	RMSE (V)	$P_{0.25}$ (%)	$\nu_{p}$ (mm/s)
11.4	A	10,000	30.000	11.439	0.102	0.109	94.31	1.004
11.4	B	10,000	30.000	11.439	0.092	0.100	95.57	0.942
12.2	A	10,000	30.000	12.131	0.123	0.141	98.38	2.892
12.2	B	10,000	30.000	12.122	0.123	0.146	99.02	2.830
13.2	A	10,000	30.000	13.259	0.094	0.111	94.40	1.175
13.2	B	10,000	30.000	13.245	0.077	0.089	97.27	0.811

## Data Availability

The data supporting the findings of this study are available from the corresponding author upon reasonable request and with permission from the affiliated organization.

## Abbreviations

AVC	Arc-voltage control
GTAW	Gas tungsten arc welding
KF	Kalman filter
AKF	Adaptive Kalman filter
EEI	Effective-error identification
FGA	Fuzzy-PID gain adaptation
MAE	Mean absolute error
NSGA-II	Non-dominated sorting genetic algorithm II
PID	Proportional–integral–derivative
SSE	Steady-state error
RMSE	Root-mean-square error
TIG	Tungsten inert gas
